# Supplementary material for: Comparison of diagnostic performance between convolutional neural networks and human endoscopists for diagnosis of colorectal polyp: A systematic review and meta-analysis
Source: PLoS One. 2021 Feb 16;16(2):e0246892. doi: 10.1371/journal.pone.0246892 (PMC7886136; doi:10.1371/journal.pone.0246892)
Supplement: S1 Table — (DOC) [file pone.0246892.s001.doc]

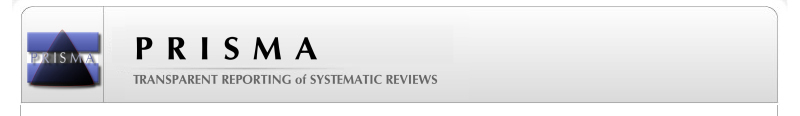
**PRISMA 2009 Flow Diagram**

**Screening**

**Included**

**Eligibility**

**Identification**

102 in PubMed

44 in EMBASE

31 in Web of Science

12 in Cochrane Library

Additional articles identified through reference list

(n = 5)

Records after duplicates removed
(n =43)

Records screened
(n =43)

Records excluded (n =15)

Non-English publications (n=3);

Case-reports (n=1);

Reviews (n=11).

Full-text articles assessed for eligibility
(n =28)

Full-text articles excluded (n =15):

Precise data unavailable (n=10);

Irrelevant subjects (n=5).

Studies included in qualitative synthesis
(n =13)

Studies included in quantitative synthesis (meta-analysis)
(n = 13)
