# Supplementary material for: Comparison of diagnostic performance between convolutional neural networks and human endoscopists for diagnosis of colorectal polyp: A systematic review and meta-analysis
Source: PLoS One. 2021 Feb 16;16(2):e0246892. doi: 10.1371/journal.pone.0246892 (PMC7886136; doi:10.1371/journal.pone.0246892)
Supplement: S3 Table — (DOCX) [file pone.0246892.s003.docx]

**S3 Table. Subgroup analysis without the data of short or full videos in the field of CP detection.**

| **Object** | **Sensitivity (95% CI)** | **Specificity**  **(95% CI)** | **PLR**  **(95% CI)** | **NLR**  **(95% CI)** | **DOR**  **(95% CI)** | **SROC**  **(95% CI)** |
| --- | --- | --- | --- | --- | --- | --- |
| **CNN** | 0.878 [ 0.702-0.956] | 0.968 [0.945-0.981 | 27.314 [14.985-49.788] | 0.126 [0.047-0.338] | 216.250  [ 53.307-877.255] | 0.98  [ 0.97-0.99] |

CNN: convolutional neural networks; DOR: diagnostic odds ratio; NLR: negative likelihood ratio; PLR: positive likelihood ratio; SROC: summary receive operating characteristic.
