# Supplementary material for: Comparison of diagnostic performance between convolutional neural networks and human endoscopists for diagnosis of colorectal polyp: A systematic review and meta-analysis
Source: PLoS One. 2021 Feb 16;16(2):e0246892. doi: 10.1371/journal.pone.0246892 (PMC7886136; doi:10.1371/journal.pone.0246892)
Supplement: Attachment — Submitted filename: PONE-D-20-22694_R1_reviewer (1).pdf [file pone.0246892.s006.pdf]

PLOS ONE

Comparison of diagnostic performance between convolutional neural networks and human endoscopists for diagnosis of colorectal polyp: a systematic review and meta-analysis

--Manuscript Draft--

**Manuscript Number:**	PONE-D-20-22694R1
**Article Type:**	Research Article
**Full Title:**	Comparison of diagnostic performance between convolutional neural networks and human endoscopists for diagnosis of colorectal polyp: a systematic review and meta-analysis
**Short Title:**	Comparison of diagnostic performance between convolutional neural networks and human endoscopists
**Corresponding Author:**	Xuezhong Xu<br>Wujin Hospital Affiliated to Jiangsu University<br>Changzhou, CHINA
**Keywords:**	Artificial intelligence; Convolutional Neural Networks; endoscopists; Diagnosis; colorectal polyp; meta-analysis
**Abstract:**	Prospective randomized trials and observational studies have demonstrated that early detection, classification, and removal of neoplastic colorectal polyp (CP) can greatly improve the prevention of colorectal cancer (CRC). Currently, the effectiveness of the diagnostic performance of colonoscopy is far from satisfactory with unstable accuracy. The convolutional neural networks (CNN) system based on artificial intelligence (AI) technology has shown its potential to assist endoscopists in increasing diagnostic accuracy. However, there are still several limitations of the CNN system, and controversies exist regarding whether it can provide better diagnostic performance than human endoscopists. Therefore, the present study was conducted to address this issue. Online databases (PubMed, Web of Science, Cochrane Library, and EMBASE) were used to search for studies done up to April 2020. The quality assessment of diagnostic accuracy scale-2 (QUADAS-2) was used to evaluate the quality of the included studies. The pretest and post-test probabilities were conducted using Fagan's nomogram. Moreover, publication bias was assessed using the Deeks' funnel plot. Thirteen studies were enrolled for this meta-analysis (ranged from 2016 to 2020). In the field of CP detection, the pooled sensitivity/specificity was 90.9%/97.8%. In the present study, it was shown that the diagnostic performance of the CNN system was comparable to that of an expert but better than that of a non-expert. Meanwhile, in the field of CP classification, the pooled sensitivity and specificity was 93.5%/77.0%. The diagnostic performance of the CNN system was superior to that of the expert and non-expert. From the results of the study, it was shown that the sensitivity, specificity, and accuracy of the CNN system were higher than that of both the experts and non-experts. Therefore, the CNN system had a satisfactory diagnostic performance for CP and could be used as a potential clinical diagnostic tool to assist endoscopists during colonoscopy.
**Order of Authors:**	Yixin Xu
	Yibo Wang
	Yulin Tan
	Cheng Xi
	Nianyuan Ye
	Dapeng Wu
	Xuezhong Xu
**Response to Reviewers:**	Dear Editor:<br>Thank you and the reviewers for your valuable suggestions. We have carefully read through the comments and made proper revisions. Our responses to the reviewer's questions are listed below. We greatly appreciated your time and efforts to improve our

Powered by Editorial Manager® and ProduXion Manager® from Aries Systems Corporation

manuscript for publication.
Sincerely,
Yixin Xu

Journal Requirements:
When submitting your revision, we need you to address these additional requirements.

1. Please ensure that your manuscript meets PLOS ONE's style requirements, including those for file naming. The PLOS ONE style templates can be found at https://journals.plos.org/plosone/s/file?id=wjVg/PLOSOne_formatting_sample_main_body.pdf and https://journals.plos.org/plosone/s/file?id=ba62/PLOSOne_formatting_sample_title_authors_affiliations.pdf

Response:
We have revised the manuscript following the style requirements.

2. Please include the date(s) on which you accessed the databases or records to obtain the data used in your study.

Response:
We completed the data collection on April 30, 2020. It was mentioned on Page 6, Line 117.

3. Thank you for quantifying study heterogeneity.
Please provide more detailed reporting on your results, for example, by reporting your Q or I^2 statistics.

Response:
In the field of CP detection, we add the value of I2 and P for CNN system on Page 14, Line 248-249; for expert on Page 15-16, Line 274-276; for non-expert on Page 16, Line 282-284.
In the field of CP classification, we add the value of I2 and P for CNN system on Page 16, Line 289-290; for expert on Page 17, Line 309-310; for non-expert on Page 17, Line 314-315.
All these data added were marked in green.

4. We note that you state in your manuscript "The design and protocol of the present study were approved by our institutional review board."
Since this is a systematic review and does not include non-public data, this statement is not necessary and may be taken out.
If you would like to keep this statement, please provide the full name of your ethics committee and the ethics approval number.

Response:
We have taken out this statement “The design and protocol of the present study were approved by our institutional review board.”, and added “As the data in this study was from previous studies, there was no need to get the ethics committee approval, follow the Declaration of Helsinki, or have patients informed consent form.” to the Ethical approval part.

5. PLOS requires an ORCID iD for the corresponding author in Editorial Manager on papers submitted after December 6th, 2016. Please ensure that you have an ORCID iD and that it is validated in Editorial Manager. To do this, go to ‘Update my Information’ (in the upper left-hand corner of the main menu), and click on the Fetch/Validate link next to the ORCID field. This will take you to the ORCID site and allow you to create a new iD or authenticate a pre-existing iD in Editorial Manager. Please see the following video for instructions on linking an ORCID iD to your Editorial Manager account: https://www.youtube.com/watch?v=_xcclfuvtxQ

Response:
We have added the ORCID to the Editorial Manager.

6. Please include captions for your Supporting Information files at the end of your

manuscript, and update any in-text citations to match accordingly. Please see our Supporting Information guidelines for more information: http://journals.plos.org/plosone/s/supporting-information

Response:
We have added the supporting information at the end of the manuscript and cited on Page 10, Line 206.

Reviewer 1#
This is an interesting systematic review article written by Xu et al. This review is well-written and described using appropriate statistical methods. The authors conducted meta-analysis to compare the diagnostic performance of artificial intelligence (AI) for detecting and classifying the colorectal polyp to the human endoscopists. This study must be of interest to the reader and will provide useful knowledge to the reader. I have one comment for the authors.

1. Shouldn't the classification of polyps be divided into normal observation, magnification (80×) and super magnification (520×, microscopic observation) for analysis? Please revisit and consider this point.

Response:
Thanks a lot for your summary and kind remarks.
To your constructive suggestion, we have carefully reviewed all the references in our meta-analysis. We found that only few studies included in our analysis mentioned the magnification type of their images used to train the CNN system. Only Kudo et al. and Mori et al. used the super magnification (520×). As a result, we did not divide the classification into different groups. Besides, we found that the classification system of CNN applied in colonoscopy mainly focused on determining whether the polyps, especially those diminutive polyps, were hyperplastic or neoplastic. For neoplastic polyps, simple resection may be not sufficient. Endoscopic Mucosal Resection (EMR), Endoscopic submucosal dissection (ESD) or even surgery is the optimal curative option. The process of classification of polyps were finished using the routine colonoscopy images (some systems using narrow-band images). Besides, magnification colonoscopy is currently not commercially available worldwide.

Reviewer 2#
The meta-analysis presented by Dr Xu and colleagues addresses an interesting topic, that is the performance of convolutional neural networks for detection and classification of colorectal polyps.
The fact that computer-aided colonoscopy and artificial intelligence are hot-topics is demonstrated by the very recent publication of other dedicated systematic reviews and meta-analysis, such as Barua et al, Endoscopy 2020 or Hassan et al, GIE 2020 (detection) and Lui et al, GIE 2020 (detection and classification).
In this meta-analysis, the authors only focus of CNNs, a specific type of artificial intelligence.
Unfortunately, the paper presented by dr. Xu has some important limitations:

Response:
Thank you for this summary of our paper.
I started my manuscript on January 10, 2020. Before that, I have not found any similar article through Searching PubMed and other database. I thought this subject about artificial intelligence applied in the field of colonoscopy was interesting and “hot”. I finished my manuscript on June, and submitted it to PLOS ONE on July 22. I have not realized that there were three articles about this subject have already been published. Those limitations you mentioned were addressed below.

1. The search strategy seems well designed, and I appreciated the effort to analyze the quality of included studies and the possibility of publication bias. However, at least one recent full-study about CNNs that met the inclusion criteria was not included in the analysis (Urban 2018, Gastroenterology); also some abstracts (such as Misawa 2019 GIE, Matsui 2019 GIE, LUI 2019 GIE) were not included. The choice whether to include or not abstracts can be discussed (even if in a meta-analysis they are usually included), however abstracts were not included in the exclusion criteria.

Powered by Editorial Manager® and ProduXion Manager® from Aries Systems Corporation

Response:
Urban 2018, Gastroenterology was already included in my analysis. I marked it in red in Table 1 and Table 2. It is also the number 30 in my reference list (in red).
We have carefully read these abstracts (Misawa 2019 GIE, Matsui 2019 GIE, LUI 2019 GIE) and we found that they could not provide precise data for analysis. As a result, we decided not to include them. For more accurate expression, we added “abstracts” to the exclusion criteria part and marked it in red (Page 6, Line 132).

2. Data extraction from the selected studies was not so rigorous and clear.
For example, apparently for reference 13 (Renner et al) authors have included the performances of CNN considering either high-confidence predictions or "standard" predictions (see table 2), with potential duplication of data and overestimation of performance.

Response:
Thank you for this constructive suggestion. We carefully reviewed the article of Renner et al., and found maybe the high-confidence predictions had the potential of duplication of data. As a result, we decided to delete the data of high-confidence, and recalculated the whole part of classification. There are so many changes, and all of them were marked in red. (Page 16, Line 288, 289, 291, 292, 294, 295; Page 17, Line 307-309; Page 19, Line 346). Table 4, Table 6, Fig. 5, Fig. 6, Fig. 8, and Fig. 9 were remade. Besides, we added some notes to Fig. 3 (Page 15, Line 261-264), and Fig. 5 (Page 17, Line 298-299). All these changes were marked in red.

3. Moreover, for reference 25 (Guo et al) the authors considered per-frame sensitivity and specificity of short and long videos; however, in order to provide clinically useful information (similar to polyp or adenoma detection rates) per-video sensitivity and specificity (reported in table 3 of the cited paper for the 100 short videos) should have been used instead. In table 3 of the paper by Guo et al are also reported the diagnostic performances of 2 experts e 2 non-experts physicians that were not included in the metanalysis.

Response:
There are two reasons that we did use per-frame sensitivity and specificity instead of per-video sensitivity and specificity. First, almost of the articles included in our analysis used colonoscopy images instead of videos to compare the diagnostic performance between the CNN system and human endoscopists. In order to ensure the consistency of the whole analysis, we chose the per-frame sensitivity and specificity. Second, for per-video sensitivity and specificity, Guo et al. did not provide enough data for us to analyze. In table 3 of their paper, they only provide the data of per-video sensitivity, but not the data of per-video specificity. As a result, we cannot get the data of (TP, TN, FP, and FN).
When we read the paper of Guo et al., we have found that they reported the diagnostic performance of experts and non-experts. But the data of human endoscopists are per-video, not per-frame. Based on the reasons we mentioned above, we decided to use the data of per-frame instead of per-video. So we did not include the data of human endoscopists.

4. Similarly, for Wang et al (ref 31) the 1633 polyps (and not the total number of polyps images) should have been considered.

Response:
The total 1633 polyps belong to Dataset A and C.
In the paper of Wang et al., they used four databases to validate the diagnostic performance of CNN system. Dataset A and B contained colonoscopy images. Dataset C and D contained colonoscopy video. However, they only provide precise data of Dataset A. As a result, we chose included the data of Dataset A. When we search for the references, we have found almost all authors used colonoscopy images to compare diagnostic performance between CNN system and human endoscopists. So we decided to choose colonoscopy images as the main source of our analysis.

5. I think that the use of the Fagan Nomogram to calculate post-test probability considering positive and negative likelihood ratios is not really indicated in this field.

Powered by Editorial Manager® and ProduXion Manager® from Aries Systems Corporation

Response:
The Fangan Nomogram is based on Bayes' Theorem. It is often used to evaluate the clinical application of a diagnostic method or tool. The pre-test probability is calculated using Bayes' Theorem. The post-test probability of LR-positive means the probability of patients suspected of this disease would get a positive diagnostic result using this diagnostic method or tool. While the post-test probability of LR-negative means the probability of patients who are not suspected of this disease would suffer it after this diagnostic method or tool gives them a negative diagnostic result.
After carefully thinking about the meaning of Fangan Nomogram, we think the application of it in the field of detection and classification of colorectal polyps may be appropriate.

6. The differential performance according to dimension of polyps (diminutive and non-diminutive polyps) should have been analysed

Response:
Sometimes colorectal polyps are hard to detect and classify, especially for the diminutive ones. So maybe the diagnostic performance would be different between diminutive polyps and non-diminutive polyps. After carefully reviewed all the references, we found that seven studies provided data about diminutive polyps (Chen et al., Renner et al., Mori et al., Ozawa et al., Shin et al., Byrne et al., and Kudo et al.). Among them, Kudo et al. focused on the detection of Polyps, and Chen et al., Mori et al., and Byrne et al. mainly focused on diminutive polyps. Because only one study provided data about diminutive polyps in the detection part, so we thought it was not necessary to perform a subgroup analysis. In the subgroup analysis of diminutive polyps in the classification part, the result showed the performance of the CNN and expert had just slightly changed. Probably, the reason is because only two studies (Renner et al., and Ozawa et al.) provided data about non-diminutive polyps. After careful consideration, we decided not to add the subgroup analysis to the manuscript, due to the slight change of the results and the consistency of the article.
The subgroup analysis was shown below:
ObjectSensitivity (95% CI)Specificity
(95% CI)PLR
(95% CI)NLR
(95% CI)DOR
(95% CI)SROC
(95% CI)
CNN 0.935 [0.915-0.947]0.770 [0.597-0.883]4.061 [2.155-7.652]0.085 [0.060-0.120]47.849 [18.638-122.842]0.983 [0.951-0.997]
Expert0.912 [0.827-0.957]0.848 [0.756-0.910]6.009 [3.783-9.544]0.104 [0.054-0.200]57.970 [30.407-110.518]0.938 [0.923-0.953]
Non-expert0.878 [0.792-0.932]0.782 [0.687-0.854]3.915 [2.673-5.731]0.174 [0.112-0.267]25.428 [13.169-49.096]0.895 [0.873-0.924]
Diminutive polyps
CNN0.948 [0.925-0.965]0.812 [0.673-0.900]5.037 [2.741-9.258]0.064 [0.040-0.101]79.210 [29.347-213.791]0.965 [0.941-0.972]
Expert0.903 [0.808-0.954]0.864 [0.777-0.921]4.659 [3.099-8.818]0.112 [0.057-0.221]49.389 [30.057-117.346]0.921 [0.901-0.965]
Non-expert0.878 [0.792-0.932]0.782 [0.687-0.854]3.915 [2.673-5.731]0.174 [0.112-0.267]25.428 [13.169-49.096]0.895 [0.873-0.924]
Note: The sample size of non-diminutive polyps is too small, so it cannot be analyzed. The data of non-expert in whole group are identical to those in diminutive polyps group, so the results are just the same.

7. The paper requires a deep linguistic revision (grammatical mistakes, repetitions…), possibly by an English mother-tongue revisor.

Response:
We have already sent this paper to language polishing. (All changes were marked in blue.)

Other observations:
8. Reference 10 does not refer to detection of colonic lesions, but of early esophageal squamous cell carcinoma/preneoplastic lesions in the esophagus. The sentence in the

Powered by Editorial Manager® and ProduXion Manager® from Aries Systems Corporation

introduction related to this reference should be revised.

Response:
We are sorry for this mistake. We have revised this mistake and cited another reference.

9. Both table 1 and 2 should contain the reference numbers of the included papers in order to make the tables easier to read. Moreover, I suggest to list the papers according to the year of publication.

Response:
We added the reference number to Table 1 and 2 and listed papers according to year of publication. Besides, I fixed two references' names and marked them in red. (Kuo is altered to Kudo; Yu is altered to Lequan)

10. Table 1 should also include more details about the imaging modalities included in the different studies. For example, the use of dedicated endocytoscopes in the studies referred as 12 and 25 should be reported. Also information about the use of real-time analysis by the CNN systems, and the type of lesions included (only diminutive polyps or polyps of any size) in the different studies should be highlighted in the table.

Response:
We have added information of type of endocytoscopes, type of CNN system, real-time use of CNN system, type of lesions, and type of images into Table 1, and marked them in red.

11. In the paper it is stated that 7 of the included studies focused on detection; however in table 1 only 6 papers have detection been reported as field focus; similarly, 5 studies should include experts human endoscopists but only four papers are reported in table 1 and 2.

Response:
Actually, there are 7 studies focused on detection, they are Lequan et al., Wang et al., Urban et al., Zhang et al., Yamada et al., Kudo et al., and Guo et al.. In fact, we made a mistake. There are only 4 studies provided data about experts and 3 studies provided data about non-experts. We revised it and marked it in red. (Page 10, Line 216)

12. The paper by Yu (2016), included in table 1 and 2 is not reported in the references.

Response:
Yu (2016) is actually Lequan (2016), we just mistook his/her last name. We have revised it and marked it in red.

Review 3#
1. There are some spelling mistakes:
- line 186, page 15: Results instead of resules
- line 211, page 17, table 2: proximal rectosigmoid instead of poximal rectosigmoid
- line 360, page 25: ordinary physicians instead of phyisicians

Response:
We have revised all the spelling mistakes, and already sent the manuscript to language polishing.

2. There are missing the technical explanations of the different CNN-systems you compared in your analysis: Why are some CNN-systems better for CP detection and others for CP classification? Is it justified to compare different CNN-systems that provide different features? Do they have different characteristics concerning deep learning?

Response:
For CP detection training, authors chose images contained CP and those did not contain CP. Their purpose is to train the CNN system to distinguish CP from normal colorectal mucosa. This task is also very difficult. Because flat, small and isochromatic

Powered by Editorial Manager® and ProduXion Manager® from Aries Systems Corporation

	polyps are associated with a high miss rate, even for experts.<br>While for CP classification training, authors used images contained hyperplastic or neoplastic CP. The authors used NBI or magnification images to train CNN system to classify different pathological type of CP. For hyperplastic CP, the treatment is just simple resection. While for neoplastic CP, it is not enough. Endoscopic Mucosal Resection (EMR), Endoscopic submucosal dissection (ESD) or even surgery is the optimal curative option. If the accuracy of classification function of CNN system is high enough, endoscopists can trust the diagnostic results provided by it and make a simultaneous therapeutic decision while colonoscopy.<br>Maybe the details of algorithms of detection and classification are slightly different. But rare authors provided information about these differences. However, they are all based on CNN basically.<br><br>3. There is also missing the description of the different classification-systems of CP used by different authors in different studies.<br><br>Response:<br>We have added information of different type of CNN system used in different studies in Table 1.
**Additional Information:**	
**Question**	**Response**
**Financial Disclosure**<br><br>Enter a financial disclosure statement that describes the sources of funding for the work included in this submission. Review the submission guidelines for detailed requirements. View published research articles from *PLOS ONE* for specific examples.<br><br>This statement is required for submission and **will appear in the published article** if the submission is accepted. Please make sure it is accurate.	The author(s) received no specific funding for this work.

**Unfunded studies**<br>Enter: *The author(s) received no specific funding for this work.*<br><br>**Funded studies**<br>Enter a statement with the following details:<br>• Initials of the authors who received each award<br>• Grant numbers awarded to each author<br>• The full name of each funder<br>• URL of each funder website<br>• Did the sponsors or funders play any role in the study design, data collection and analysis, decision to publish, or preparation of the manuscript?<br>• **NO** - Include this sentence at the end of your statement: *The funders had no role in study design, data collection and analysis, decision to publish, or preparation of the manuscript.*<br>• **YES** - Specify the role(s) played.<br><br>* typeset	
**Competing Interests**<br><br>Use the instructions below to enter a competing interest statement for this submission. On behalf of all authors, disclose any competing interests that could be perceived to bias this work—acknowledging all financial support and any other relevant financial or non-financial competing interests.<br><br>This statement **will appear in the published article** if the submission is accepted. Please make sure it is accurate. View published research articles from *PLOS ONE* for specific examples.	The authors have declared that no competing interests exist.

**NO authors have competing interests**<br><br>Enter: *The authors have declared that no competing interests exist.*<br><br>**Authors with competing interests**<br><br>Enter competing interest details beginning with this statement:<br><br>*I have read the journal's policy and the authors of this manuscript have the following competing interests: [insert competing interests here]*<br><br>* typeset	
**Ethics Statement**<br><br>Enter an ethics statement for this submission. This statement is required if the study involved:<br><br>• Human participants<br>• Human specimens or tissue<br>• Vertebrate animals or cephalopods<br>• Vertebrate embryos or tissues<br>• Field research<br><br>Write "N/A" if the submission does not require an ethics statement.<br><br>General guidance is provided below. Consult the submission guidelines for detailed instructions. **Make sure that all information entered here is included in the Methods section of the manuscript.**	N/A

**Format for specific study types**<br><br>**Human Subject Research (involving human participants and/or tissue)**<br>• Give the name of the institutional review board or ethics committee that approved the study<br>• Include the approval number and/or a statement indicating approval of this research<br>• Indicate the form of consent obtained (written/oral) or the reason that consent was not obtained (e.g. the data were analyzed anonymously)<br><br>**Animal Research (involving vertebrate animals, embryos or tissues)**<br>• Provide the name of the Institutional Animal Care and Use Committee (IACUC) or other relevant ethics board that reviewed the study protocol, and indicate whether they approved this research or granted a formal waiver of ethical approval<br>• Include an approval number if one was obtained<br>• If the study involved *non-human primates*, add *additional details* about animal welfare and steps taken to ameliorate suffering<br>• If anesthesia, euthanasia, or any kind of animal sacrifice is part of the study, include briefly which substances and/or methods were applied<br><br>**Field Research**<br><br>Include the following details if this study involves the collection of plant, animal, or other materials from a natural setting:<br>• Field permit number<br>• Name of the institution or relevant body that granted permission	
**Data Availability**<br><br>Authors are required to make all data underlying the findings described fully available, without restriction, and from the time of publication. PLOS allows rare exceptions to address legal and ethical concerns. See the PLOS Data Policy and FAQ for detailed information.	Yes - all data are fully available without restriction

Powered by Editorial Manager® and ProduXion Manager® from Aries Systems Corporation

A Data Availability Statement describing where the data can be found is required at submission. Your answers to this question constitute the Data Availability Statement and **will be published in the article**, if accepted.<br><br>**Important:** Stating 'data available on request from the author' is not sufficient. If your data are only available upon request, select 'No' for the first question and explain your exceptional situation in the text box.<br><br>Do the authors confirm that all data underlying the findings described in their manuscript are fully available without restriction?	
**Describe where the data may be found in full sentences. If you are copying our sample text, replace any instances of XXX with the appropriate details.**<br><br>• If the data are **held or will be held in a public repository**, include URLs, accession numbers or DOIs. If this information will only be available after acceptance, indicate this by ticking the box below. For example: *All XXX files are available from the XXX database (accession number(s) XXX, XXX.*).<br>• If the data are all contained **within the manuscript and/or Supporting Information files**, enter the following: *All relevant data are within the manuscript and its Supporting Information files.*<br>• If neither of these applies but you are able to provide **details of access elsewhere**, with or without limitations, please do so. For example:<br><br>*Data cannot be shared publicly because of [XXX]. Data are available from the XXX Institutional Data Access / Ethics Committee (contact via XXX) for researchers who meet the criteria for access to confidential data.*<br><br>*The data underlying the results presented in the study are available from (include the name of the third party*	All relevant data are within the manuscript and its Supporting Information files.

and contact information or URL).<br>• This text is appropriate if the data are owned by a third party and authors do not have permission to share the data.<br><br>* typeset	
Additional data availability information:	

Powered by Editorial Manager® and ProduXion Manager® from Aries Systems Corporation

Manuscript Click here to access/download;Manuscript;Manuscript.docx

Comparison of diagnostic performance between convolutional neural networks and human endoscopists for diagnosis of colorectal polyp: a systematic review and meta-analysis

Yixin Xu[1], Yibo Wang[1], Yulin Tan[1], Cheng Xi[1], Nianyuan Ye[1], Dapeng Wu[2], Xuezhong Xu[1*]

[1] Department of General Surgery, Changzhou Wujin People's Hospital Affiliated to Jiangsu University; The Wujin Clinical College of Xuzhou Medical University, Changzhou, Jiangsu Province, China.

[2] Department of Endoscopy, Jiangsu Provincial Hospital of Traditional Chinese Medicine, Nanjing, Jiangsu, China.

***** Corresponding author

Email: xxz197001@sina.com

Abstract

Prospective randomized trials and observational studies have demonstrated that early detection, classification, and removal of neoplastic colorectal polyp (CP) can greatly improve the prevention of colorectal cancer (CRC). Currently, the effectiveness of the diagnostic performance of colonoscopy is far from satisfactory with unstable accuracy. The convolutional neural networks (CNN) system based on artificial intelligence (AI) technology has shown its potential to assist endoscopists in increasing diagnostic accuracy. However, there are still several limitations of the CNN system, and controversies exist regarding whether it can provide better diagnostic performance than human endoscopists. Therefore, the present study was conducted to address this issue. Online databases (PubMed, Web of Science, Cochrane Library, and EMBASE) were used to search for studies done up to April 2020. The quality assessment of diagnostic accuracy scale-2 (QUADAS-2) was used to evaluate the quality of the included studies. The pretest and post-test probabilities were conducted using Fagan's nomogram. Moreover, publication bias was assessed using the Deeks' funnel plot. Thirteen studies were enrolled for this meta-analysis (ranged from 2016 to 2020). In the field of CP detection, the pooled sensitivity/specificity was 90.9%/97.8%. In the present study, it was shown that the diagnostic performance of the CNN system was comparable to that of an expert but better than that of a non-expert. Meanwhile, in the field of CP classification, the pooled sensitivity and specificity was 93.5%/77.0%. The diagnostic performance of the CNN system was superior to that of the expert and non-expert. From the results

of the study, it was shown that the sensitivity, specificity, and accuracy of the CNN system were higher than that of both the experts and non-experts. Therefore, the CNN system had a satisfactory diagnostic performance for CP and could be used as a potential clinical diagnostic tool to assist endoscopists during colonoscopy.

Introduction

In 2018, it was reported that colorectal cancer (CRC) had approximately 1,800,000 new cases and 881,000 deaths, implying 1 in 10 cancer cases and deaths [1]. About 85% of CRCs were shown to have developed from precancerous polyps through genetic and epigenetic ways with a mean dwell time of at least ten years [2, 3]. Hence, early and accurate detection of colorectal polyp (CP) has great significance for the prevention of CRC. Colonoscopy is the most effective and essential method in early diagnosis and prevention of CRC through detection and removal of the neoplastic lesion before its progression to invasive cancer [4]. It was reported that at least 70% of CRCs could be prevented with regular colonoscopies and removal of polyps [4]. Therefore, it’s of great importance to achieve better diagnostic accuracy of CP for their prevention and better treatment.

Pathologically, CP can be categorized into inflammatory polyp sessile, hyperplastic polyp, serrated adenoma polyp (SSAP), and adenoma [5]. The risk of developing CRC is different for each classification. Several studies have shown that adenoma, similar to SSAP, has the highest risk for development and progression to CRC. In contrast, it is rare for hyperplastic and inflammatory polyp to develop to CRC [5, 6]. Therefore, how to accurately classify CP is important for both the endoscopists and patients, since precise differentiation of CP results in the reduction of unnecessary endoscopic resection which subsequently decreases the incidence of surgical complications, medical costs and labor burden of doctors [7].

Though, colonoscopy is effective in the early diagnosis of CRC, it is far from

being perfect and has several important limitations. Firstly, it has a relatively-high missed diagnosis rate [8]. Secondly, some neoplastic lesions are difficult to detect, even for expert endoscopists [9]. In addition, the task is time-consuming for the endoscopists and labor-intensive which can result in higher costs, especially in countries with large populations. Lastly, the diagnostic performance of colonoscopy highly depends on the working experience of endoscopists, which varies among individuals. This implies that the diagnostic accuracy of colonoscopy is unstable.

To overcome these shortcomings, several studies have reported the use of artificial intelligence to improve medical diagnosis. They are shown as follows. Recently, convolutional neural networks (CNN) have shown significant potential to assist endoscopists leading to increased diagnostic accuracy of CP during colonoscopy [10]. It is a type of the most prevalent network architectures of deep learning (DL) methods based on artificial intelligence (AI) technology. Moreover, findings from some studies showed that the CNN system could automatically classify CP, which is significantly helpful for the therapeutic decision-making process during colonoscopy [11-13]. However, this technology is still immature and far from satisfactory. Moreover, a majority of controversies exist regarding whether the CNN system can provide a better performance than the human endoscopists, and whether it is worthy of popularizing.

Therefore, the present study was conducted to compare the diagnostic performance between the CNN system and human endoscopists in the field of CP detection and classification.

Materials and Methods

Literature search strategy

A systematic literature search was conducted online for studies assessing the diagnostic value of the CNN system applied in the field of colonoscopy for colorectal polyp detection and classification. PubMed, Web of Science, Cochrane Library, and EMBASE (up to April 30, 2020) were searched with the combination of the following terms: ([“artificial intelligence”] OR [“convolutional neural networks”] OR [“deep learning”] OR [“computer-aided”]) AND ([“colonoscopy”] OR [“endoscopy”]) AND ([“colon”] OR [“rectum”] OR [“colonic”] OR [“rectal”] OR [“colorectal”]) AND ([“polyp”] OR [“polyps”]).

All article sections were carefully reviewed. Subsequently, we screened bibliographies of the retrieved articles to identify any potential source of relevant studies.

Study selection

The inclusion criteria included: (1) studies included patients with CP; (2) colonoscopy was performed to detect or classify colorectal polyps; (3) CNN system was applied to improve the diagnostic performance of colonoscopy; (4) precise diagnostic data were presented in the article; (5) if the colorectal polyps were classified, the final pathology results were provided. The exclusion criteria included: (1) the types of articles were abstracts, reviews, letters, comments, and case reports; (2) precise data were unavailable in the article; (3) animal studies and non-English

publications.

Data extraction and quality assessment

Two independent researchers (Ye and Xi) conducted the data extraction from the included studies. The information of enrolled studies included the first author's name, publication year, country, diseases concerned, training material, testing material, types of diagnostic performance, and diagnostic performance of the CNN system, expert, and non-expert. The diagnostic performance was categorized as true-positive (TP), false-positive (FP), true-negative (TN), and false-negative (FN) and was retrieved from each article. Moreover, if there was any inconsistency between the two reviewers (Ye and Xi), a discussion was conducted by a third investigator to resolve the problem.

The methodological quality and applicability of the studies included were evaluated using the quality assessment of diagnostic accuracy scale-2 (QUADAS-2) [14].

Outcomes of interests

First, pooled sensitivity, specificity, and other diagnostic indexes were calculated according to the value of TP, FP, TN, and FN, among CNN system, expert, and non-expert. Secondly, the diagnostic odds ratio (DOR) and the area (AUC) under the summary receive operating characteristic (SROC) curve, which represent overall diagnostic performance, were examined and compared among different groups. To

identify whether the differences of diagnostic performance were statistically significant, the relative diagnostic odds ratio (RDOR) was compared between each of the two groups (CNN system vs. expert; CNN system vs. non-expert; expert vs. non-expert.

Statistical analyses

Statistical analyses were performed to determine the diagnostic efficacy. The sensitivity, specificity, positive likelihood ratio (PLR), negative likelihood ratio (NLR), DOR, the AUC of SROC, and RDOR were pooled with their 95% confidence interval (CI). A diagnostic tool was considered to have strong diagnostic value, if its PLR was above 5 and NLR was below 0.2 [15]. The heterogeneity among studies was evaluated by Cochran Q and Higgins' I^2 statistics [16]. If the value of I^2 was more than 50%, and the value of P less than 0.05, indicating statistically significant heterogeneity existed, then a random-effect model was selected for pooling the data [17]. Otherwise, a fixed-effect model was utilized.

SROC was estimated based on the Moses-Littenberg method [18]. According to the AUC of SROC, the overall diagnostic performance was categorized into four levels, including reasonable (<0.75), good (0.75–0.92), very good (0.93–0.96), and excellent (≥ 0.97) [19].

The pretest probability was defined as the prevalence of the target condition. Meanwhile, the clinical utility of a diagnostic test was evaluated using the post-test probability, which was based on Bayes theorem [20]. They were calculated using the

likelihood ratio (LR). Post-test probability = LR × pretest probability / ([1-pretest probability] × [1-LR]) [21]. The pretest and post-test probabilities were shown in the Fagan's nomogram.

RDOR compared between each two groups to identify statistically significant differences of diagnostic performance and was based on multivariate meta-regression analysis [22, 23]. The Deeks' funnel plot was used to assess the publication bias.

Pooled sensitivity, specificity, accuracy, PLR, NLR, DOR, and AUC of SROC were calculated using Stata version 14.0. QUADAS-2 assessment was performed using Review Manager version 5.3, while he SROC curves were conducted using Meta-Disc version 1.4. The result was considered to be statistically significant, if *P*-value was below 0.05.

Ethical approval

As the data in this study was from previous studies, there was no need to get the ethics committee approval, follow the Declaration of Helsinki, or have patients informed consent form.

Results

Search strategy

Following the initial search through the different databases, 189 articles were identified (102 in PubMed, 31 in Web of Science, 12 in Cochrance Library, and 44

in EMBASE). Firstly, 146 duplicate studies were removed. Subsequently, the remaining 43 articles were screened after duplicates were removed. Fifteen articles, including non-English publications, reviews, abstracts, and case reports, which did not meet the inclusion criteria were rules out. Subsequently, those articles with imprecise data and irrelevant subjects were excluded after full-text articles were assessed. Finally, thirteen articles were included in the present meta-analysis (Fig 1). PRISMA flow diagram and checklist were shown in S1 and S2 Tables, respectively.

Fig 1. Flow chart of studies identified, excluded and included.

Cohort characteristics and quality of included studies

Finally, 13 studies were enrolled in this meta-analysis [10-13, 24-32]. Seven of the studies included focused on the field of CP detection, while other studies focused on the field of CP classification. In those included studies, 5 studies were performed in Japan, 4 in China, 1 in Germany, one in the USA, 1 in Norway, and 1 in Canada, respectively. All articles were published in the last four years (Table 1). Meanwhile, all studies included precise data about the diagnostic performance of the CNN system; 4 studies provided precise data about performance of expert, and 3 studies provided precise data about the performance of non-expert. Histological examination results were the golden standard in the studies done about CP classification. The diagnostic performance was categorized as TP, FP, FN, and TN (Table 2).

Table 1. Characteristics of the studies included.

**Author**	**Year**	**Country**	**type of endocytoscopes**	**type of CNN system**	**real-time use of CNN system**	**type of lesions**	**type of images**	**Training material**	**Testing material**	**Field focused**	**Testing objects**
**Lequan[27]**	2016	China	images from online database	3D fully convolutional neural networks	N/A	polyps of any size	N/A	Images	Images	Detection	CNN
**Byrne[24]**	2017	Canada	190 series colonoscopes (Olympus)	deep convolutional neural networks	Yes	diminutive polyps	NBI	Videos	Videos	Classification	CNN
**Chen[11]**	2018	China	CF-H260AZI, PCF-Q260AZI,CF-HQ290AZI (Olympus)	N/A	N/A	diminutive polyps	NBI	Images	Images	Classification	CNN/Expert/ Non-expert
**Wang[10]**	2018	China	Olympus Evis Lucera CV260 (SL)/CV290 (SL) and Fujinon 4400/4450 HD	N/A	Yes	polyps of any size	N/A	Images	Images	Detection	CNN
**Renner[13]**	2018	Germany	Olympus Evis Exera III CF－HQ 190 colonoscopes	computer-assisted optical biopsy	N/A	polyps of any size	WLI/NBI	Images	Images	Classification	CNN/Expert
**Mori[12]**	2018	Japan	CFH290ECI colonoscopes (Olympus)	N/A	Yes	diminutive polyps	NBI and methylene blue staining modes	Images	Images	Classification	CNN/Expert/ Non-expert
**Shin[29]**	2018	Norwa	images from	N/A	N/A	polyps of any	N/A	Images	Images	Classification	CNN

		y	online database			size					
**Urban[30]**	2018	USA	PCF-H190 colonoscopes(Olympus)	VGG16,VGG19,and ResNet50	Yes	polyps of any size	N/A	Images	Images	Detection	CNN
**Zhang[32]**	2018	China	images from online database	ResYOLO	Yes	polyps of any size	N/A	Images/	Images	Detection	CNN
**Yamada[31]**	2019	Japan	images from online database	Faster R-CNN with VGG16	Yes	polyps of any size	N/A	Images	Images	Detection	CNN
**Kudo[26]**	2019	Japan	CF-H290ECI(Olympus)	EndoBRAIN	N/A	polyps of any size	WLI/NBI	Images	WLI/NBI images	Detection	CNN/Expert/ Non-expert
**Guo[25]**	2020	Japan	Fujinon 4450 HD	YOLOv3	Yes	polyps of any size	WLI/NBI	Images	Short/full videos	Detection	CNN
**Ozawa[28]**	2020	Japan	Evis Lucera and CF Type H260AL/I, PCF Type Q260AI, Q260AZI, H290I, and H290ZI (Olympus)	Single Shot MultiBox Detector	N/A	polyps of any size	WLI/NBI	Images	Images	Classification	CNN

CNN: convolutional neural networks; NBI: Narrow band imaging; ResYOLO: residual learning modules based on YOLO; YOLO: a CNN system named you only look once; WLI: White light imaging;

Table 2. Diagnostic performance of CNN system, expert, and non-expert.

Author	Different grouping standard	CNN system				Expert				Non-expert			
		**TP**	**FP**	**TN**	**FN**	**TP**	**FP**	**TN**	**FN**	**TP**	**FP**	**TN**	**FN**
**Lequan[27]**		3062	414	9260	1251								
**Byrne[24]**		104	2	19	4								
**Chen[11]**	Group 1	181	21	75	7	183	22	74	5	183	29	67	5
	Group 2					184	33	63	4	176	33	63	12
	Group 3									154	22	74	34
	Group 4									158	11	85	30
**Wang[10]**		6404	881	20691	2345								
**Renner[13]**	Neoplastic	48	18	30	3	48	12	36	3				
	Diminutive rectosigmoid	8	6	21	0	38	9	39	14				
**Mori[12]**	Proximal-rectosigmoid	167	9	21	12	300	12	48	58	278	20	40	80
	Rectosigmoid	95	6	135	5	176	14	268	24	161	30	252	39
**Urban[30]**		7127	83	1203	228								
**Zhang[32]**		3087	398	13057	1226								
**Yamada[31]**		732	41	4094	20								
**Kudo[26]**	WLI	1260	0	700	40	603	20	330	20	920	40	460	380
	Diameter<5mm	960	0	680	40	453	20	320	47	690	236	444	310
	NBI	1260	40	660	40	608	12	338	42	807	100	600	493
	Diameter<5mm	960	40	640	40	459	12	328	41	578	97	583	422
**Guo[25]**	Short videos	2112	642	21692	1608								
	Full videos	37938	5590	78658	5672								
**Ozawa[28]**		1073	175	74	99								
	Diameter<10mm	969	171	174	84								

CNN: convolutional neural networks; FN: false-negative; FP: false-positive; NBI: narrow band imaging; TN: true negative; TP: true positive; WLI: white light imaging.

The quality of all thirteen studies included was considered moderate, according the to QUADAS-2 assessment (Fig 2). Eleven studies were considered high quality with low risk in at least five of the seven QUADAS-2 domain. For the patient selection domain, two studies introduced bias because case-control design was avoided [24, 28]. Three studies were considered to have a high concern for

applicability [11, 26, 28]. Subsequently, for the index test domain, two studies had a high risk of bias [11, 26]. Finally, there was only one study that had a high concern regarding reference standard applicability [12].

Fig 2. Methodological quality of the included 13 studies using assessment tool of QUADAS-2. (**A**) Grouped bar charts of risk of bias (left) and concerns for applicability (right). (**B**) Quality assessment for each individual study. QUADAS-2 = Quality Assessment of Diagnostic Accuracy Studies-2.

Application in the field of colorectal polyp detection

Diagnostic performance of CNN system

The results of the diagnostic performance of the CNN system are shown in Fig 3. The pooled sensitivity and specificity were 0.909 (95% CI: 0.836-0.95) and 0.978 (95% CI: 0.949-0.991), respectively. The heterpgeneity of the sensitivity (I^2=99.93, *P*=0.00) and specificity (I^2=99.87, *P*=0.00) are significant. In addition, the pooled PLR, NLR, and DOR were 42.248 (95% CI: 37.272-56.840), 0.093 (95% CI: 0.061-0.135), 208.943 (95% CI: 143.761-303.682), respectively. The AUC of SROC of the CNN system was 0.983 (95% CI: 0.971-0.992). The results are as shown in Table 3. Moreover, the PLR and NLR results of the CNN system showed that it is a good method for detecting colorectal polyps. As a result, we conducted the Fagan nomogram to evaluate the clinical application of the CNN system. The results suggested that the post-test probability of a positive result was 91% if a pretest

probability was 20%. Meanwhile, for a negative test result, a NLR of 0.09 reduced the post-test probability to 2% (Fig 4).

Fig 3. The pooled diagnostic accuracy index of CNN system in the field of CP detection. (**A**) Sensitivity, (**B**) specificity. a: short videos; b:full videos; c: WLI; d: WLI/diameter smaller than 5 mm; e: NBI; f: NBI/diameter smaller than 5mm. CNN: convolutional neural networks; CP: colorectal polyps. NBI: narrow-blue images; WLI: white-light images.

Fig 4. Fagan nomogram for CNN system in the field of CP detection. CNN: convolutional neural networks; CP: colorectal polyps.

Table 3. Diagnostic performance of CNN system, expert, and non-expert in the field of colorectal polyp detection.

Object	Sensitivity (95% CI)	Specificity (95% CI)	PLR (95% CI)	NLR (95% CI)	DOR (95% CI)	SROC (95% CI)
**CNN**	0.909 [0.836-0.954]	0.978 [0.949-0.991]	42.248 [37.272-56.840]	0.093 [0.061-0.135]	208.943 [143.761-303.682]	0.983 [0.971-0.992]
**Expert**	0.936 [0.906-0.957]	0.954 [0.941-0.964]	39.772 [24.901-46.235]	0.072 [0.046-0.099]	306.923 [179.061-526.082]	0.986 [0.952-0.983]
**Non-expert**	0.651 [0.598-0.700]	0.840 [0.734-0.909]	4.162 [2.203-7.871]	0.432 [0.352-0.524]	9.76 [4.853-19.634]	0.734 [0.712-0.790]

CNN: convolutional neural networks; DOR: diagnostic odds ratio; NLR: negative likelihood ratio; PLR: positive likelihood ratio; SROC: summary receive operating characteristic.

Diagnostic performance of expert and non-expert

As shown in Table 3, the pooled sensitivity and specificity of expert were 0.936 (95%CI: 0.906-0.957) and 0.954 (95%CI: 0.941-0.964). The heterogeneity of sensitivity is significant (I^2=85.09, *P*=0.00), and the heterogeneity of specificity is

moderate (I^2=30.37, *P*=0.23). Additionally, the PLR, NLR, DOR and AUC of SROC were 39.772 (95%CI: 24.901-46.235), 0.072 (95%CI: 0.046-0.099), 306.923 (95%CI: 179.061-526.082), and 0.986 (95%CI: 0.952-0.983), respectively.

Subsequently, for non-expert, the pooled sensitivity, specificity, PLR, NLR, DOR and AUC of SROC were 0.651 (95%CI: 0.598-0.700), 0.840 (95%CI: 0.734-0.909), 4.162 (95%CI: 2.203-7.871), 0.432 (95%CI: 0.352-0.524), 9.76 (95%CI: 4.853-19.634), and 0.7335 (95%CI: 0.712-0.790), respectively. The heterogeneity of sensitivity (I^2=94.42, *P*=0.00) and specificity (I^2=98.30, *P*=0.00) are significant.

Application in the field of colorectal polyp classification

Diagnostic performance of CNN system

First, the pooled sensitivity and specificity were 0.935 (95%CI: 0.915-0.947) and 0.770 (95%CI: 0.597-0.883) (Fig 5). The heterogeneity of sensitivity (I^2=96.28, *P*=0.00) and specificity (I^2=97.92, *P*=0.00) are significant. Meanwhile, the PLR, NLR, DOR, and AUC of SROC were 4.061 (95%CI: 2.155-7.652), 0.085 (95%CI: 0.060-0.120), 47.849 (95%CI: 18.638-122.842), and 0.983 (95%CI: 0.951-0.997), respectively. The Fagan nomogram suggested that the post-test probability of a positive result was 50%, if a pretest probability was 20%. Besides, for a negative test result, a NLR of 0.08 reduced the post-test probabilty to 2% (Fig 6).

Fig 5. The pooled diagnostic accuracy indexes of CNN system in the field of CP

classification. (**A**) Sensitivity, (**B**) specificity. a: neoplastic; b: diminutive rectosigmoid; c: rectosigmoid; d: proximal-rectosigmoid; CNN: convolutional neural networks; CP: colorectal polyps.

Fig 6. Fagan nomogram for CNN system in the field of CP classification. CNN: convolutional neural networks; CP: colorectal polyps.

Diagnostic performance of expert and non-expert

For the diagnostic performance of expert in the field of classification of colorectal polyps, the pooled sensitivity, specificity, PLR, NLR, DOR and AUC of SROC of expert were 0.912 (95%CI: 0.827-0.957), 0.848 (95%CI: 0.756-0.910), 6.009 (95%CI: 3.783-9.544), 0.104 (95%CI: 0.054-0.200), 57.970 (95%CI: 30.407-110.518), and 0.938 (95%CI: 0.923-0.953), respectively. The heterogeneity of sensitivity (I^2=88.17, *P*=0.00) and specificity (I^2=87.62, *P*=0.00) are significant.

Besides, the pooled sensitivity, specificity, PLR, NLR, DOR and AUC of SROC of non-expert were 0.878 (95%CI: 0.792-0.923), 0.782 (95%CI: 0.687-0.854), 3.915 (95%CI: 2.673-5.731), 0.174 (95%CI: 0.112-0.267), 25.428 (95%CI: 13.169-49.096), and 0.895 (95%CI: 0.873-0.924), respectively. The heterogeneity of sensitivity (I^2=91.38, *P*=0.00) and specificity (I^2=88.75, *P*=0.00) are significant.

All data is summarized in Table 4.

Table 4. Diagnostic performance of CNN system, expert, and non-expert in the field of colorectal polyp classification.

**Object**	**Sensitivity (95% CI)**	**Specificity (95% CI)**	**PLR (95% CI)**	**NLR (95% CI)**	**DOR (95% CI)**	**SROC (95% CI)**

**CNN**	0.935 [0.915-0.947]	0.770 [0.597-0.883]	4.061 [2.155-7.652]	0.085 [0.060-0.120]	47.849 [18.638-122.842]	0.983 [0.951-0.997]
**Expert**	0.912 [0.827-0.957]	0.848 [0.756-0.910]	6.009 [3.783-9.544]	0.104 [0.054-0.200]	57.970 [30.407-110.518]	0.938 [0.923-0.953]
**Non-expert**	0.878 [0.792-0.932]	0.782 [0.687-0.854]	3.915 [2.673-5.731]	0.174 [0.112-0.267]	25.428 [13.169-49.096]	0.895 [0.873-0.924]

CNN: convolutional neural networks; DOR: diagnostic odds ratio; NLR: negative likelihood ratio; PLR: positive likelihood ratio; SROC: summary receive operating characteristic.

The comparison of diagnostic performance among CNN system, expert, and non-expert

As shown in Fig 7, the performance of the expert was the best in the detection of CP, followed by the CNN system, while the non-expert was the least effective. However, as compared by RDOR, the difference in diagnostic performance between the expert and CNN system was statistically insignificant (Table 5).

Moreover, for CP classification, the sequence of the superiority of diagnostic performance was the CNN system, expert, and non-expert (Fig 8). However, all the differences among CNN, expert, and non-expert were statistically insignificant (Table 6).

Fig 7. Summary receiver operation characteristic (SROC) curve of diagnostic performance of CNN system, expert, and non-expert for CP detection.

Fig 8. Summary receiver operation characteristic (SROC) curve of diagnostic performance of CNN system, expert, and non-expert for CP classification.

Table 5: Comparison of diagnostic performance among CNN, expert, and non-expert in the field of colorectal polyp detection.

Object	Coefficient	Stand error	RDOR	95% CI	P
**CNN vs. Expert**	0.011	1.1780	1.01	0.08-13.17	0.9925
**CNN vs. Non-expert**	-3.531	1.1249	0.03	0.00-0.34	0.0085
**Expert vs. Non-expert**	-3.693	0.5410	0.02	0.01-0.10	0.0010

Table 6: Comparison of diagnostic performance among CNN, expert, and non-expert in the field of colorectal polyp classification.

Object	Coefficient	Stand error	RDOR	95% CI	P
**CNN vs. Expert**	-0.153	0.6669	0.86	0.21-3.59	0.8222
**CNN vs. Non-expert**	-1.0.27	0.6621	0.36	0.08-1.51	0.1467
**Expert vs. Non-expert**	-0.767	0.4904	0.46	0.16-1.37	0.1463

Publication bias and identification of sources of heterogeneity

According to Deeks' funnel plot asymmetry, there was no publication bias in pooled results of the CNN system. For CP detection, the result was $P > |t| = 0.186$. At the same time, for CP classification, the result was $P > |t| = 0.104$. They are as shown in Figs 9A and 9B. Since notable heterogeneity was observed in the pooled analysis of the CNN system in the field of CP detection and classification, meta-regression was conducted to identify the source of heterogeneity. However, no potential sources of heterogeneity were identified.

Fig 9. Deeks' funnel plot for publication bias. (**A**) CNN system for CP detection, (**B**)

CNN system for CP classification.

Discussion

In the present study, the current status of the CNN system was systematically reviewed and applied in the field of CP detection and classification. A quantitative comparison of the diagnostic value between the CNN system and human endoscopists was made. From the results of the study, the main findings were that the diagnostic performance of the CNN system was comparable to that of an expert in the field of CP detection. In contrast, the performance of the CNN system was significantly superior to that of a non-expert. Meanwhile, in the field of CP classification, the diagnostic performance of the CNN system was better than either expert or non-expert, despite the differences were statistically insignificant.

The American Society of Gastrointestinal Endoscopy published Preservation and Incorporation of Valuable Endoscopic Innovations (PIVI) statement in 2015 to address the resect and discard strategy [33]. This strategy set the threshold of a diagnose-and-leave strategy for small colorectal polyps as NPV≥90%. At the same time, the threshold of a resect-and-discard strategy is above 90% of the agreement with histopathology for post-polypectomy surveillance intervals[34]. These set standards are very high and hard to achieve, even for experienced endoscopists. Besides, the task of endoscopists is time-consuming as well as labor-intensive. Some studies have shown that endoscopic detections and predictions led to a rather low diagnostic accuracy rate, especially in the case of non-expert use [35, 36].

Hence, the need for the application and use of technological support. This is because these studies have shown that computer-aided diagnosis of endoscopic images using AI has the potential to surpass the diagnostic accuracy of trained specialist. AI may also provide more accurate results without interobserver differences, especially between experts and non-experts.

Currently, a considerable number of studies have been done focusing on the development of the CNN system that assisted human endoscopists. In the field of colonoscopy, its function is mainly divided into two categories: detection and classification. For CP detection, we found that the DOR of the CNN system was 208.943 [95%CI: 143.761-303.682], which is lower than that of expert (306.923 [95%CI: 173.061-526.082]), but significantly better than that of non-expert (9.76 [95%CI: 4.853-19.634]). Similar results which indicated that an experienced expert had better diagnostic performance than the CNN system were also found in the field of *Helicobacter pylori* (HP) infection status evaluation [37]. However, it is important to consider that not every endoscopist has working experience for decades to qualify being an expert. Most of the endoscopists are ordinary physicians, trainees, and novices. Therefore, the application of the CNN system can significantly increase their diagnostic accuracy. Moreover, some studies showed that these non-expert endoscopists achieved a better diagnostic performance during endoscopy after the AI training course [38, 39]. Hence, the AI technologies have a potential to be applid not only as a clinical ancillary diagnostic tool, but also as an endoscopist training method.

Moreover, it would be highly beneficial if endoscopic observation can distinguish neoplastic CP from hyperplastic CP. This is because the removal of lesions without malignant potential is not only expensive, but also leads to high post-procedure complications [40]. Thus, the precise classification of CP will significantly improve the cost-effectiveness of colonoscopy. However, the task of precisely classifying the different types of CP is rather difficult. For instance, lesions with indistinct borders, flat and depressed features in conventional adenomas are challenging to distinguish from surrounding normal mucosa. This is especially common when the bowel preparation is inadequate or the mucosa is capped by mucus or intestinal residue [9]. A study conducted by Kuiper et al. showed that the sensitivity/specificity of classification of diminutive CP was only 77.0%/78.8%, which was far from satisfactory [41]. In this study, it was also shown that the sensitivity/specificity of a non-expert in the field of CP classification was 87.8%/78.2%. Thus, the benefits of optical CP classification may remain limited to experts. However, as earlier mentioned, not every endoscopist is an expert. Hence, the emergence of AI technology has greatly overcome this limitation. In the present study, we found that the diagnostic performance of the CNN system was better than both the expert and non-expert, despite the difference between the CNN system and expert being statistically insignificant. However, due to the complexity of classification technology, the DOR of the CNN system applied in the field of CP classification (47.849 [95%CI: 18.638-122.842]) was weaker than that in the field of CP detection (208.943 [95%CI: 143.761-303.682]). A similar CNN-DL system was

also used for diagnosis and classification of proximal gastric precancerous conditions, including chronic atrophic gastritis, intestinal metaplasia, and dysplasia [42]. This system achieved a sensitivity of 93.5%, a specificity of 94.0%, and an accuracy of 93.5%, which were much better than both the less and more experienced endoscopists.

However, CNN technology has several limitations.

First, most of the images and videos extracted for CNN system training are highly qualified, which usually leads to selection bias. These systems are often unable to distinguish lesions from low-quality materials. Their diagnostic performance is excellent in the training set but weak in the clinical practice.

Secondly, images and videos of rare lesions are challenging to find, such as subtle flat colonic lesions and morphology types. They are insufficient in either hospitals' independent or online databases, which will result in insufficient training of the CNN system. This will further lead to high misdiagnostic rates of infrequent diseases.

Finally, most studies included in the present review trained their CNN systems with stationary images or image frames extracted from colonoscopic videos. It may restrict the ability of real-time implementation of the CNN system. Moreover, due to the lack of calculating power of computer processors and the complexity of technical processes, the latency of the decision-making process of most systems is not satisfactory, which will subsequently disturb the endoscopist during colonoscopy. The ability to work in real-time during endoscopy should be incorporated.

With the advancements of AI technology, it can be predicted that an ideal CNN system will be developed to overcome these limitations. It may precisely distinguish different lesions from normal surrounding mucosa, including those rare lesions. Meanwhile, it may assist endoscopists simultaneously during endoscopy with almost undetectable latency. Even more, it may provide the type, location, size, depth, and other relevant information of lesions.

In the present meta-analysis study, there are some limitations to this study that should be acknowledged here. First, studies focused on this field are limited since the CNN system applied in the field of endoscopy has recently emerged and is not yet prevalent. Secondly, the sample size of comparison between the CNN system and human endoscopists is small, which may lead to selection bias. Thirdly, although there is no publication bias in the present study, since letters, reviews, as well as articles not published in English were excluded, selective reporting bias may exist. Fourthly, although meta-regression analysis was performed to identify the potential sources of heterogeneity, due to the limitation of the sample size and the variables collected from studies included, the exploration of heterogeneity may still be inadequate. Finally, a majority of studies included were retrospective and used different types of training and testing materials, which is assumed to have a potential bias.

Conclusions

In conclusion, our systematic review and meta-analysis suggested that the CNN

system achieved comparable diagnostic performance to that of an expert, but better performance than that of a non-expert, in the field of CP detection. Moreover, in the field of CP classification, the CNN system demonstrated better diagnostic performance than the human endoscopists regardless of the level of working experience. Although there are still some limitations of the CNN system, it can be popularized in clinical practice with relative-high diagnostic accuracy and thus enhance the diagnostic performance of endoscopists.

Acknowledgements

The authors thank Dr. Peng Jiang and Dr. Hai-Feng Tang for their critical reading and informative advice during the study process. Meanwhile, the authors thank Freescience for language polishing. There was no financial support received.

Author contributions

Conceptualization: Yixin Xu.

Data curation: Yixin Xu, Yulin Tan,.

Formal analysis: Dapeng Wu, Cheng Xi.

Methodology: Cheng Xi.

Software: Nianyuan Ye.

Writing – original draft: Yixin Xu, Yibo Wang.

Writing – review and editing: Xuezhong Xu.

Conflict of interest:

None of the authors have any financial interests related to this study to disclose.

Reference

1. Bray F, Ferlay J, Soerjomataram I, Siegel RL, Torre LA, Jemal A. Global cancer statistics 2018: GLOBOCAN estimates of incidence and mortality worldwide for 36 cancers in 185 countries. CA Cancer J Clin. 2018;68(6):394-424. doi: 10.3322/caac.21492. PubMed PMID: 30207593.
2. Kuntz KM, Lansdorp-Vogelaar I, Rutter CM, Knudsen AB, van Ballegooijen M, Savarino JE, et al. A systematic comparison of microsimulation models of colorectal cancer: the role of assumptions about adenoma progression. Med Decis Making. 2011;31(4):530-9. doi: 10.1177/0272989X11408730. PubMed PMID: 21673186.
3. Strum WB. Colorectal Adenomas. N Engl J Med. 2016;375(4):389-90. doi: 10.1056/NEJMc1604867. PubMed PMID: 27464213.
4. Winawer SJ, Zauber AG, Ho MN, O'Brien MJ, Gottlieb LS, Sternberg SS, et al. Prevention of colorectal cancer by colonoscopic polypectomy. The National Polyp Study Workgroup. N Engl J Med. 1993;329(27):1977-81. PubMed PMID: 8247072.
5. Ijspeert JEG, Bastiaansen BAJ, van Leerdam ME, Meijer GA, van Eeden S, Sanduleanu S, et al. Development and validation of the WASP classification system for optical diagnosis of adenomas, hyperplastic polyps and sessile serrated adenomas/polyps. Gut. 2016;65(6):963-70. doi: 10.1136/gutjnl-2014-308411. PubMed PMID: 25753029.
6. Ijspeert JEG, Bevan R, Senore C, Kaminski MF, Kuipers EJ, Mroz A, et al. Detection rate of serrated polyps and serrated polyposis syndrome in colorectal cancer screening cohorts: a European overview. Gut. 2017;66(7):1225-32. doi: 10.1136/gutjnl-2015-310784. PubMed PMID: 26911398.
7. Hassan C, Pickhardt PJ, Rex DK. A resect and discard strategy would improve cost-effectiveness of colorectal cancer screening. Clinical gastroenterology and hepatology : the official clinical practice journal of the American Gastroenterological Association. 2010;8(10). doi: 10.1016/j.cgh.2010.05.018. PubMed PMID: 20621680.
8. van Rijn JC, Reitsma JB, Stoker J, Bossuyt PM, van Deventer SJ, Dekker E. Polyp miss rate determined by tandem colonoscopy: a systematic review. Am J Gastroenterol. 2006;101(2):343-50. PubMed PMID: 16454841.
9. Yamada M, Sakamoto T, Otake Y, Nakajima T, Kuchiba A, Taniguchi H, et al. Investigating endoscopic features of sessile serrated adenomas/polyps by using narrow-band imaging with optical magnification. Gastrointest Endosc. 2015;82(1):108-17. doi: 10.1016/j.gie.2014.12.037. PubMed PMID: 25840928.
10. Wang P, Xiao X, Glissen Brown JR, Berzin TM, Tu M, Xiong F, et al. Development and validation of a deep-learning algorithm for the detection of polyps during

colonoscopy. Nat Biomed Eng. 2018;2(10):741-8. doi: 10.1038/s41551-018-0301-3. PubMed PMID: 31015647.

11. Chen PJ, Lin MC, Lai MJ, Lin JC, Lu HH, Tseng VS. Accurate Classification of Diminutive Colorectal Polyps Using Computer-Aided Analysis. Gastroenterology. 2018;154(3):568-75. doi: 10.1053/j.gastro.2017.10.010. PubMed PMID: 29042219.

12. Mori Y, Kudo SE, Misawa M, Saito Y, Ikematsu H, Hotta K, et al. Real-Time Use of Artificial Intelligence in Identification of Diminutive Polyps During Colonoscopy: A Prospective Study. Ann Intern Med. 2018;169(6):357-66. doi: 10.7326/M18-0249. PubMed PMID: 30105375.

13. Renner J, Phlipsen H, Haller B, Navarro-Avila F, Saint-Hill-Febles Y, Mateus D, et al. Optical classification of neoplastic colorectal polyps - a computer-assisted approach (the COACH study). Scand J Gastroenterol. 2018;53(9):1100-6. doi: 10.1080/00365521.2018.1501092. PubMed PMID: 30270677.

14. Whiting PF, Rutjes AWS, Westwood ME, Mallett S, Deeks JJ, Reitsma JB, et al. QUADAS-2: a revised tool for the quality assessment of diagnostic accuracy studies. Ann Intern Med. 2011;155(8):529-36. doi: 10.7326/0003-4819-155-8-201110180-00009. PubMed PMID: 22007046.

15. Deeks JJ. Systematic reviews in health care: Systematic reviews of evaluations of diagnostic and screening tests. BMJ. 2001;323(7305):157-62. PubMed PMID: 11463691.

16. Higgins JPT, Thompson SG, Deeks JJ, Altman DG. Measuring inconsistency in meta-analyses. BMJ. 2003;327(7414):557-60. PubMed PMID: 12958120.

17. Jackson D, White IR, Thompson SG. Extending DerSimonian and Laird's methodology to perform multivariate random effects meta-analyses. Stat Med. 2010;29(12):1282-97. doi: 10.1002/sim.3602. PubMed PMID: 19408255.

18. Moses LE, Shapiro D, Littenberg B. Combining independent studies of a diagnostic test into a summary ROC curve: data-analytic approaches and some additional considerations. Stat Med. 1993;12(14):1293-316. PubMed PMID: 8210827.

19. Jones CM, Athanasiou T. Summary receiver operating characteristic curve analysis techniques in the evaluation of diagnostic tests. Ann Thorac Surg. 2005;79(1):16-20. PubMed PMID: 15620907.

20. Jaeschke R, Guyatt GH, Sackett DL. Users' guides to the medical literature. III. How to use an article about a diagnostic test. B. What are the results and will they help me in caring for my patients? The Evidence-Based Medicine Working Group. JAMA. 1994;271(9):703-7. PubMed PMID: 8309035.

21. Jaeschke R, Guyatt G, Sackett DL. Users' guides to the medical literature. III. How to use an article about a diagnostic test. A. Are the results of the study valid? Evidence-Based Medicine Working Group. JAMA. 1994;271(5):389-91. PubMed PMID: 8283589.

22. Jackson D, Riley R, White IR. Multivariate meta-analysis: potential and promise. Stat Med. 2011;30(20):2481-98. doi: 10.1002/sim.4172. PubMed PMID: 21268052.

23. Ian RW. Multivariate random-effects meta-regression: Updates to mvmeta. Stata Journal. 2011;11(2):255-70. doi: 10.1177/1536867X1101100206.

24. Byrne MF, Chapados N, Soudan F, Oertel C, Linares Pérez M, Kelly R, et al.

Real-time differentiation of adenomatous and hyperplastic diminutive colorectal polyps during analysis of unaltered videos of standard colonoscopy using a deep learning model. Gut. 2019;68(1). doi: 10.1136/gutjnl-2017-314547. PubMed PMID: 29066576.

25. Guo Z, Nemoto D, Zhu X, Li Q, Aizawa M, Utano K, et al. A polyp detection algorithm can detect small polyps: An ex vivo reading test compared with endoscopists. Dig Endosc. 2020. doi: 10.1111/den.13670. PubMed PMID: 32173917.

26. Kudo SE, Misawa M, Mori Y, Hotta K, Ohtsuka K, Ikematsu H, et al. Artificial Intelligence-assisted System Improves Endoscopic Identification of Colorectal Neoplasms. Clin Gastroenterol Hepatol. 2019. doi: 10.1016/j.cgh.2019.09.009. PubMed PMID: 31525512.

27. Lequan Y, Hao C, Qi D, Jing Q, Pheng Ann H. Integrating Online and Offline Three-Dimensional Deep Learning for Automated Polyp Detection in Colonoscopy Videos. IEEE J Biomed Health Inform. 2017;21(1):65-75. doi: 10.1109/JBHI.2016.2637004. PubMed PMID: 28114049.

28. Ozawa T, Ishihara S, Fujishiro M, Kumagai Y, Shichijo S, Tada T. Automated endoscopic detection and classification of colorectal polyps using convolutional neural networks. Therap Adv Gastroenterol. 2020;13:1756284820910659. doi: 10.1177/1756284820910659. PubMed PMID: 32231710; PubMed Central PMCID: PMCPMC7092386.

29. Shin Y, Balasingham I. Automatic polyp frame screening using patch based combined feature and dictionary learning. Comput Med Imaging Graph. 2018;69:33-42. doi: 10.1016/j.compmedimag.2018.08.001. PubMed PMID: 30172091.

30. Urban G, Tripathi P, Alkayali T, Mittal M, Jalali F, Karnes W, et al. Deep Learning Localizes and Identifies Polyps in Real Time With 96% Accuracy in Screening Colonoscopy. Gastroenterology. 2018;155(4):1069-78 e8. doi: 10.1053/j.gastro.2018.06.037. PubMed PMID: 29928897; PubMed Central PMCID: PMCPMC6174102.

31. Yamada M, Saito Y, Imaoka H, Saiko M, Yamada S, Kondo H, et al. Development of a real-time endoscopic image diagnosis support system using deep learning technology in colonoscopy. Sci Rep. 2019;9(1):14465. doi: 10.1038/s41598-019-50567-5. PubMed PMID: 31594962; PubMed Central PMCID: PMCPMC6783454.

32. Zhang R, Zheng Y, Poon CCY, Shen D, Lau JYW. Polyp detection during colonoscopy using a regression-based convolutional neural network with a tracker. Pattern Recognit. 2018;83:209-19. doi: 10.1016/j.patcog.2018.05.026. PubMed PMID: 31105338; PubMed Central PMCID: PMCPMC6519928.

33. Chandrasekhara V, Desilets D, Falk GW, Inoue H, Romanelli JR, Savides TJ, et al. The American Society for Gastrointestinal Endoscopy PIVI (Preservation and Incorporation of Valuable Endoscopic Innovations) on peroral endoscopic myotomy. Gastrointest Endosc. 2015;81(5). doi: 10.1016/j.gie.2014.12.007. PubMed PMID: 25799295.

34. Rex DK, Kahi C, O'Brien M, Levin TR, Pohl H, Rastogi A, et al. The American Society for Gastrointestinal Endoscopy PIVI (Preservation and Incorporation of Valuable Endoscopic Innovations) on real-time endoscopic assessment of the histology of diminutive colorectal polyps. Gastrointest Endosc. 2011;73(3):419-22. doi: 10.1016/j.gie.2011.01.023. PubMed PMID: 21353837.
35. Rees CJ, Rajasekhar PT, Wilson A, Close H, Rutter MD, Saunders BP, et al. Narrow band imaging optical diagnosis of small colorectal polyps in routine clinical practice: the Detect Inspect Characterise Resect and Discard 2 (DISCARD 2) study. Gut. 2017;66(5):887-95. doi: 10.1136/gutjnl-2015-310584. PubMed PMID: 27196576.
36. Schachschal G, Mayr M, Treszl A, Balzer K, Wegscheider K, Aschenbeck J, et al. Endoscopic versus histological characterisation of polyps during screening colonoscopy. Gut. 2014;63(3):458-65. doi: 10.1136/gutjnl-2013-304562. PubMed PMID: 23812324.
37. Shichijo S, Endo Y, Aoyama K, Takeuchi Y, Ozawa T, Takiyama H, et al. Application of convolutional neural networks for evaluating Helicobacter pylori infection status on the basis of endoscopic images. Scandinavian journal of gastroenterology. 2019;54(2):158-63. doi: 10.1080/00365521.2019.1577486. PubMed PMID: 30879352.
38. Sehgal V, Rosenfeld A, Graham DG, Lipman G, Bisschops R, Ragunath K, et al. Machine Learning Creates a Simple Endoscopic Classification System that Improves Dysplasia Detection in Barrett's Oesophagus amongst Non-expert Endoscopists. Gastroenterol Res Pract. 2018;2018:1872437. doi: 10.1155/2018/1872437. PubMed PMID: 30245711.
39. Cai S-L, Li B, Tan W-M, Niu X-J, Yu H-H, Yao L-Q, et al. Using a deep learning system in endoscopy for screening of early esophageal squamous cell carcinoma (with video). Gastrointest Endosc. 2019;90(5). doi: 10.1016/j.gie.2019.06.044. PubMed PMID: 31302091.
40. Abu Dayyeh BK, Thosani N, Konda V, Wallace MB, Rex DK, Chauhan SS, et al. ASGE Technology Committee systematic review and meta-analysis assessing the ASGE PIVI thresholds for adopting real-time endoscopic assessment of the histology of diminutive colorectal polyps. Gastrointest Endosc. 2015;81(3). doi: 10.1016/j.gie.2014.12.022. PubMed PMID: 25597420.
41. Kuiper T, Marsman WA, Jansen JM, van Soest EJ, Haan YCL, Bakker GJ, et al. Accuracy for optical diagnosis of small colorectal polyps in nonacademic settings. Clinical gastroenterology and hepatology : the official clinical practice journal of the American Gastroenterological Association. 2012;10(9). doi: 10.1016/j.cgh.2012.05.004. PubMed PMID: 22609999.
42. Guimarães P, Keller A, Fehlmann T, Lammert F, Casper M. Deep-learning based detection of gastric precancerous conditions. Gut. 2020;69(1):4-6. doi: 10.1136/gutjnl-2019-319347. PubMed PMID: 31375599.

Supporting information

S1 Table. PRISMA flow diagram.

S2 Table. PRISMA checklist.

Fig 1 Click here to access/download;Figure;Fig 1.tif

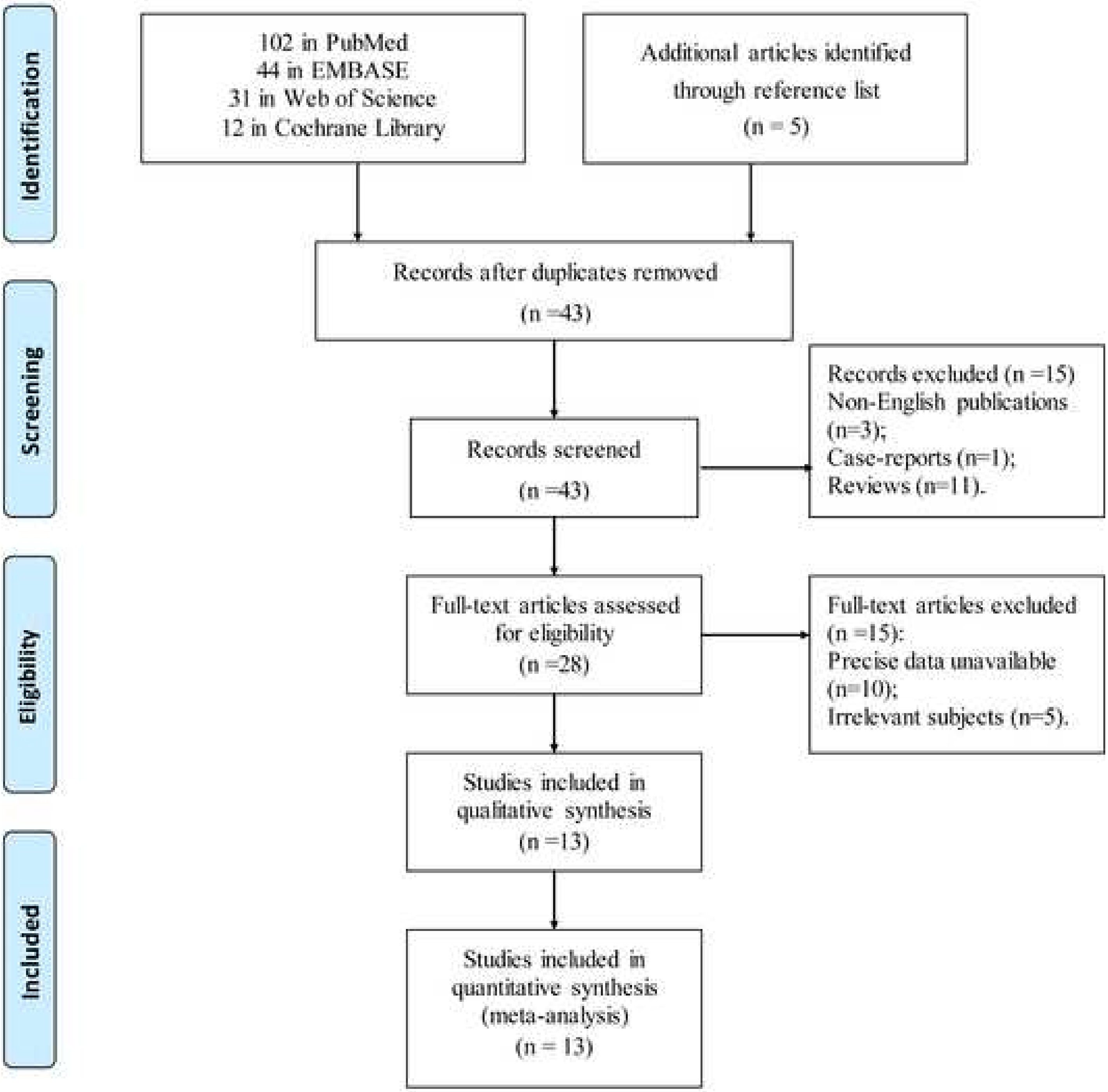

Fig 2

Click here to access/download;Figure;Fig 2.tif

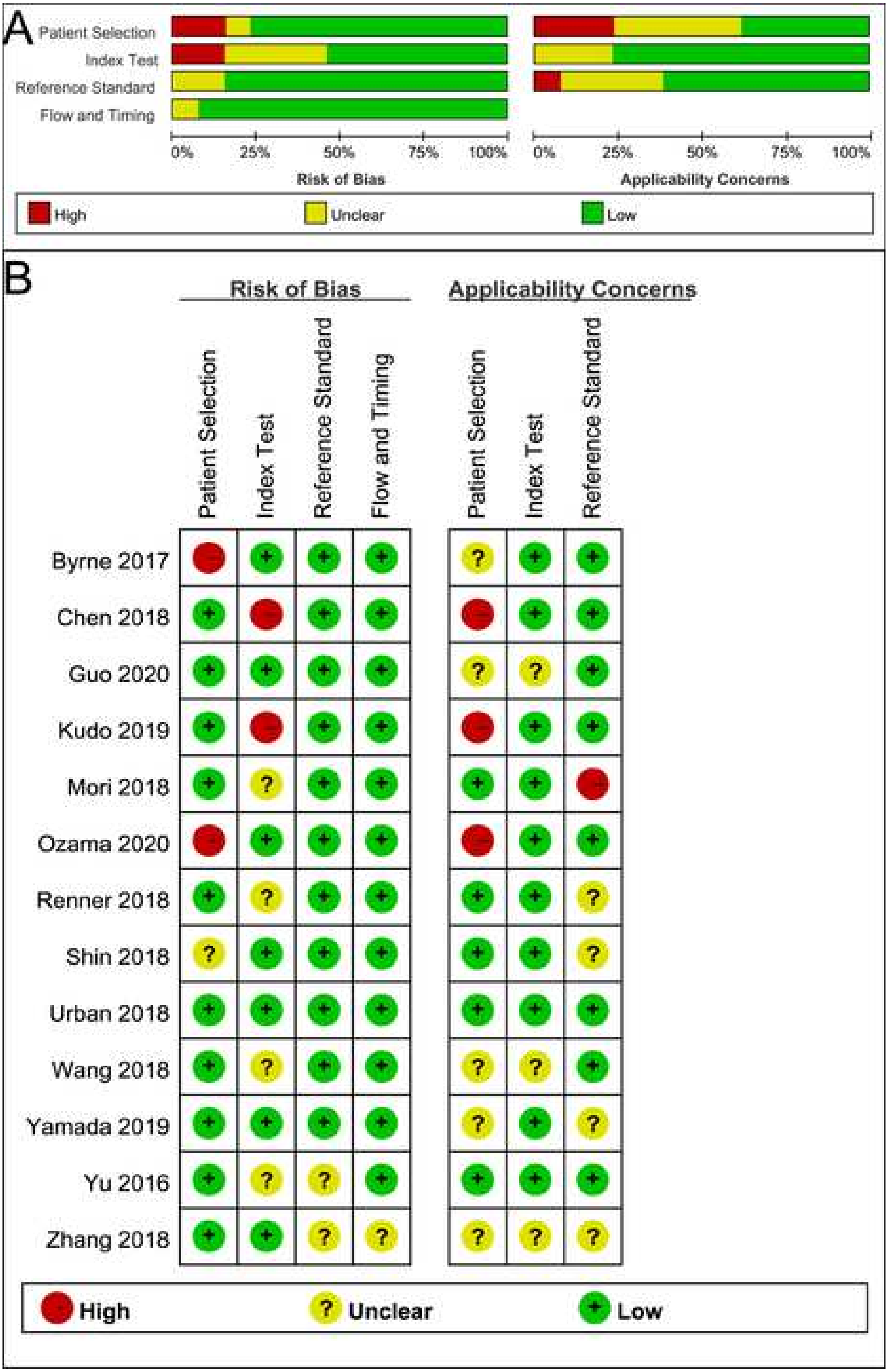

Fig 3

Click here to access/download;Figure;Fig 3.tif

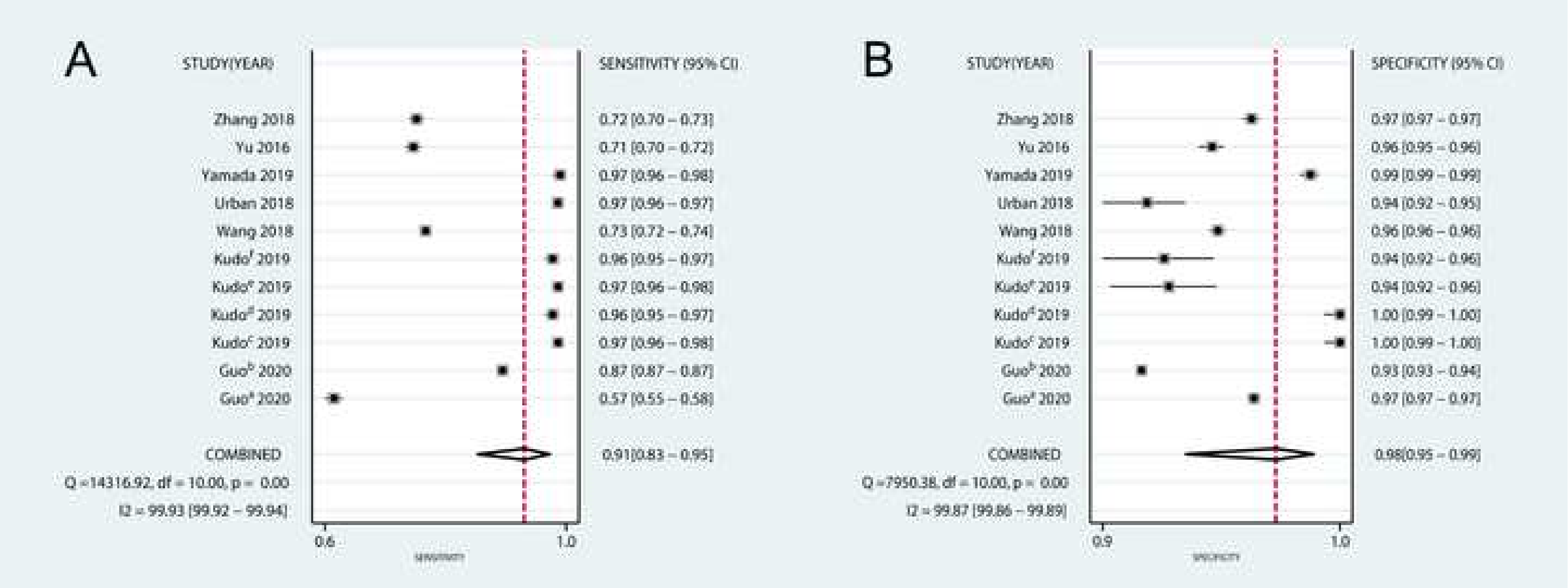

Fig 4

Click here to access/download;Figure;Fig 4.tif

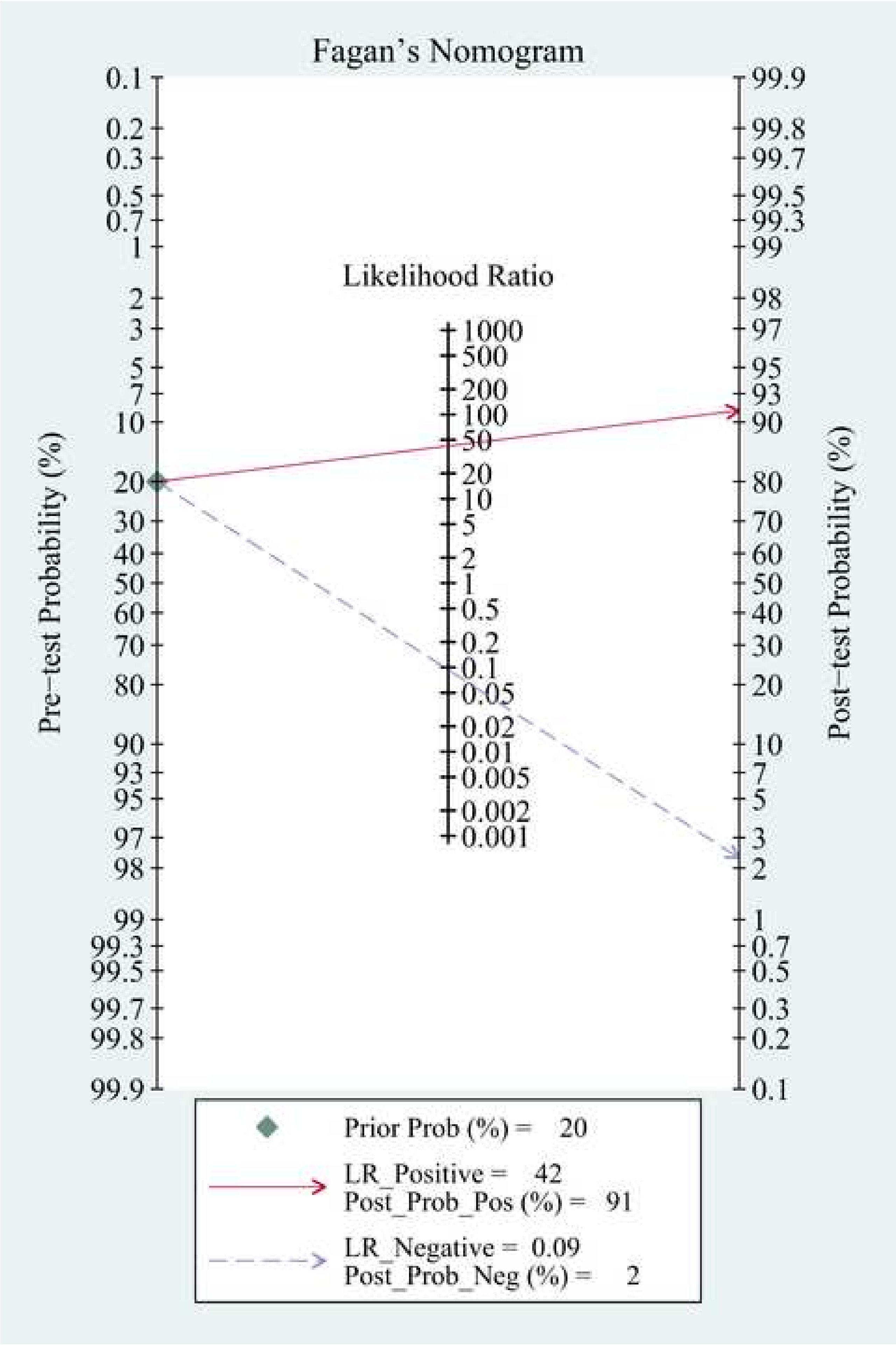

Fig 5

Click here to access/download;Figure;Fig 5.tif

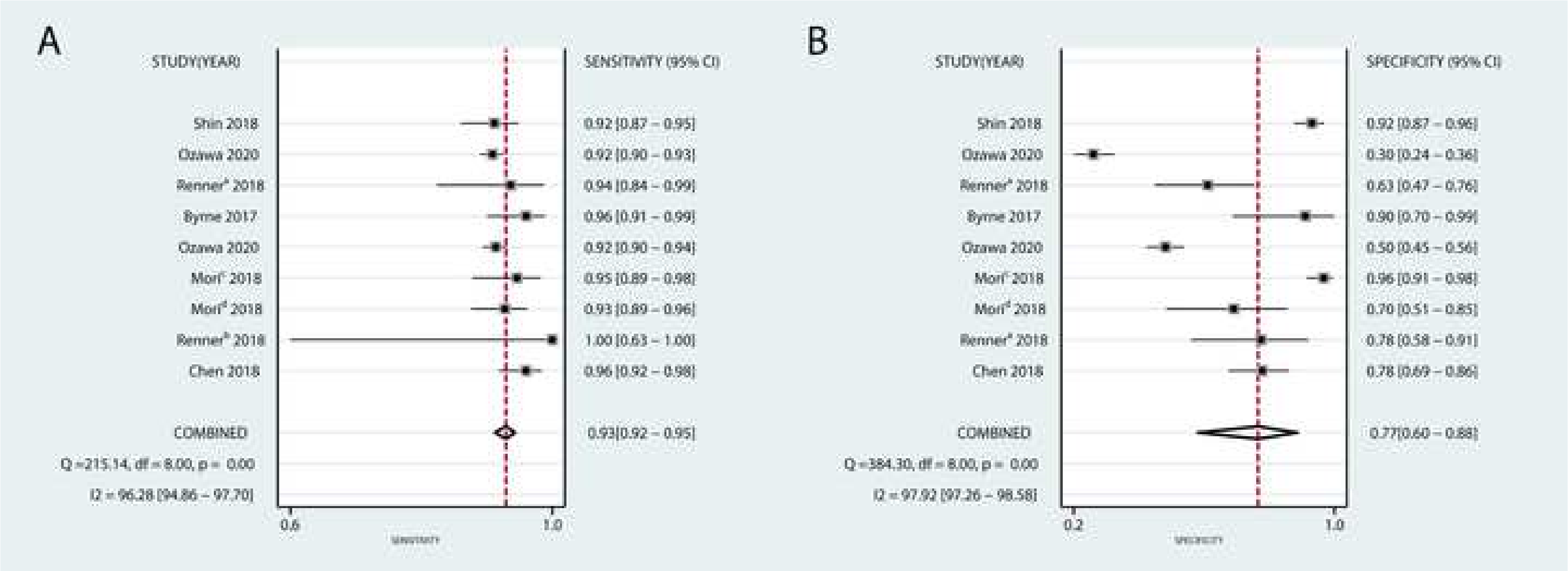

Fig 6

Click here to access/download;Figure;Fig 6.tif

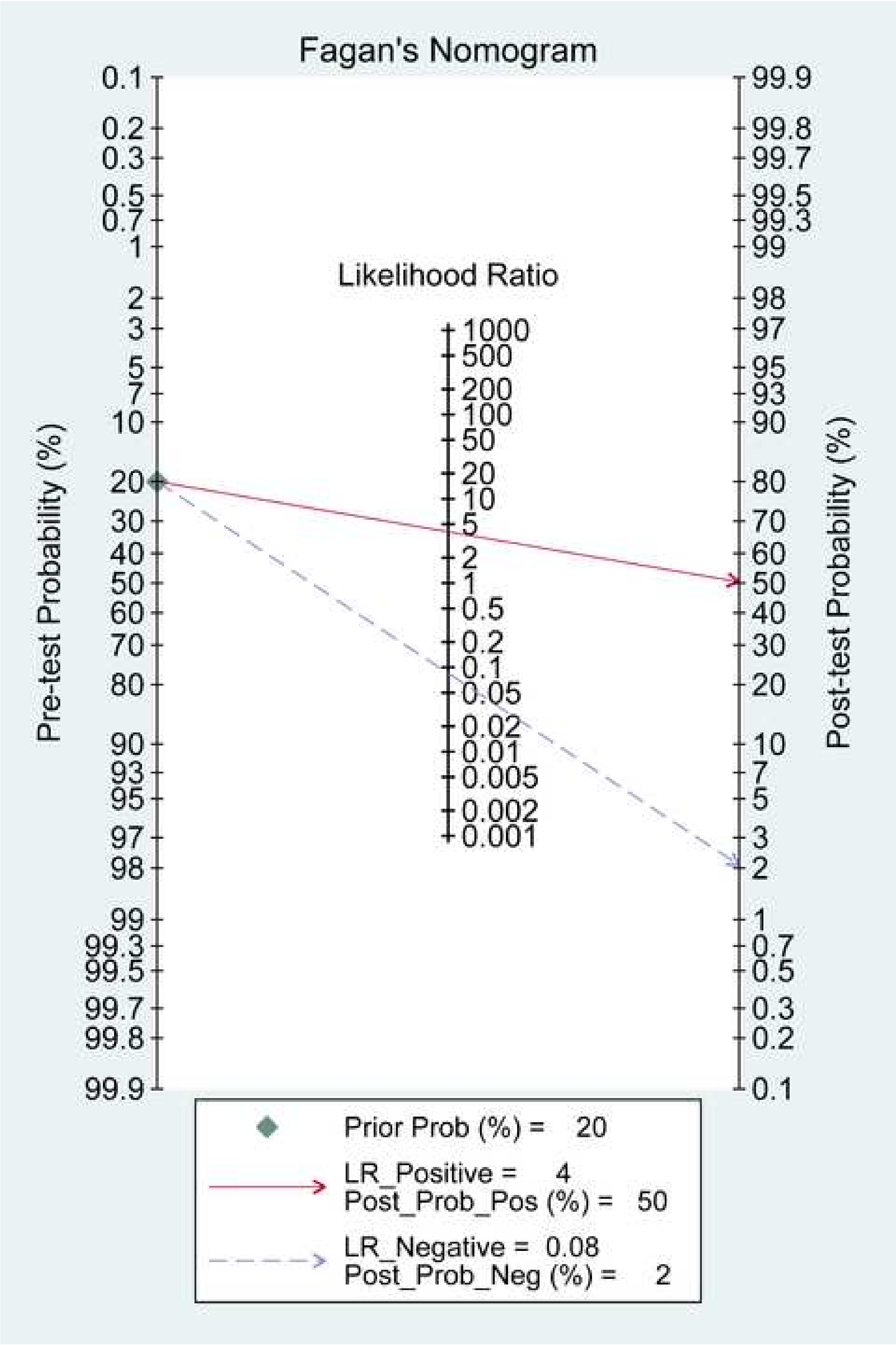

Fig 7

Click here to access/download;Figure;Fig 7.tif

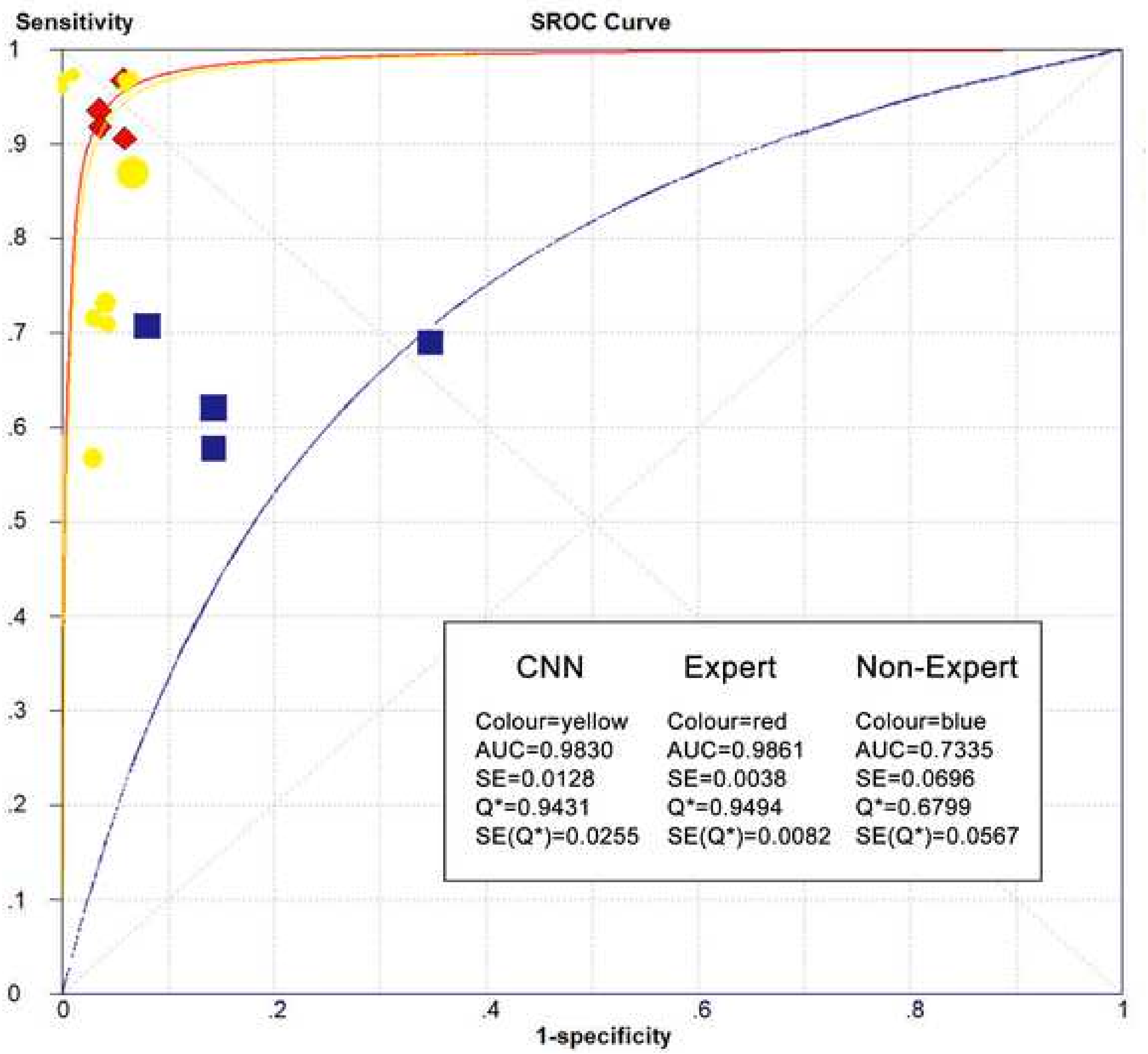

Fig 8

Click here to access/download;Figure;Fig 8.tif

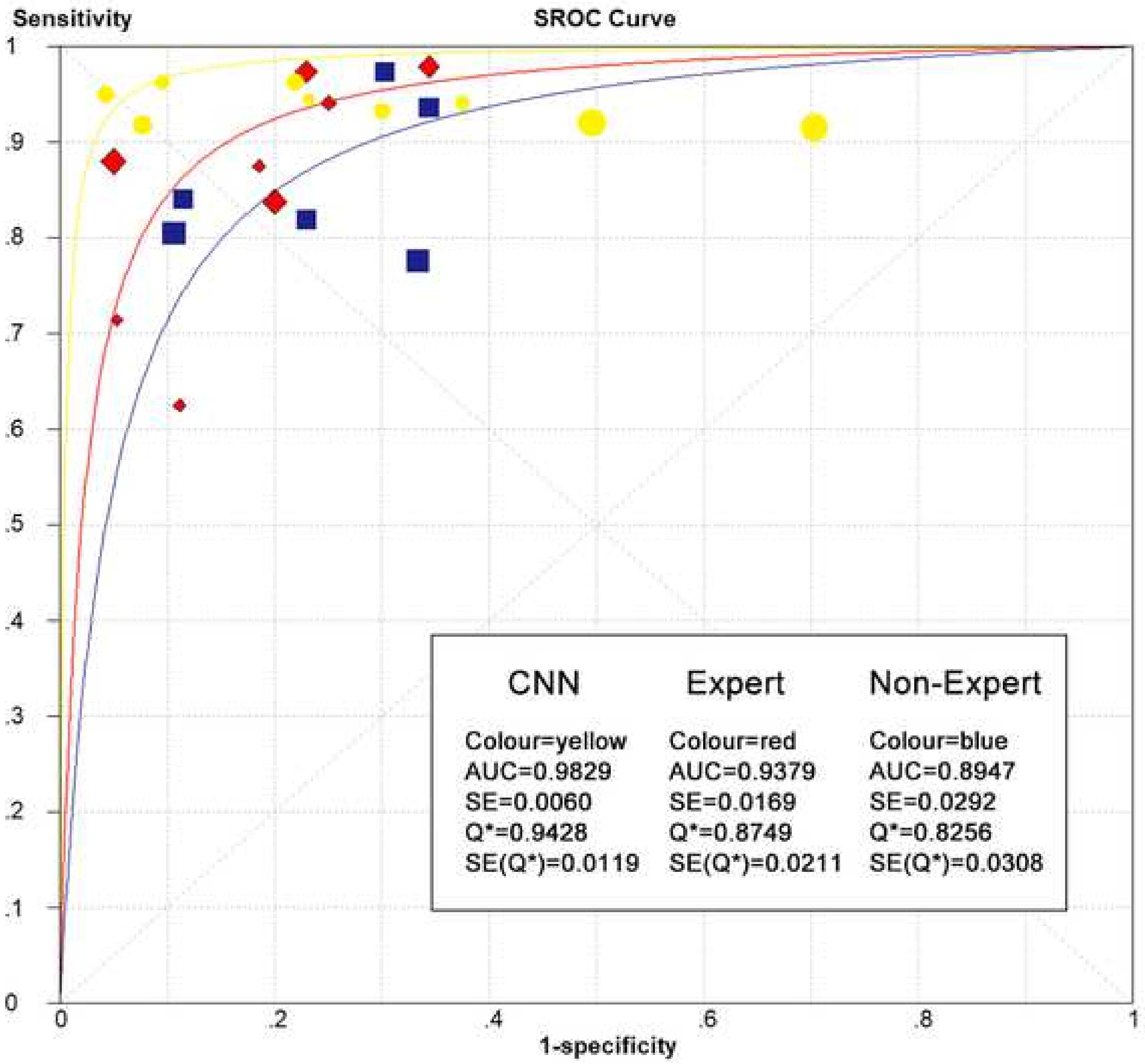

Fig 9

Click here to access/download;Figure;Fig 9.tif

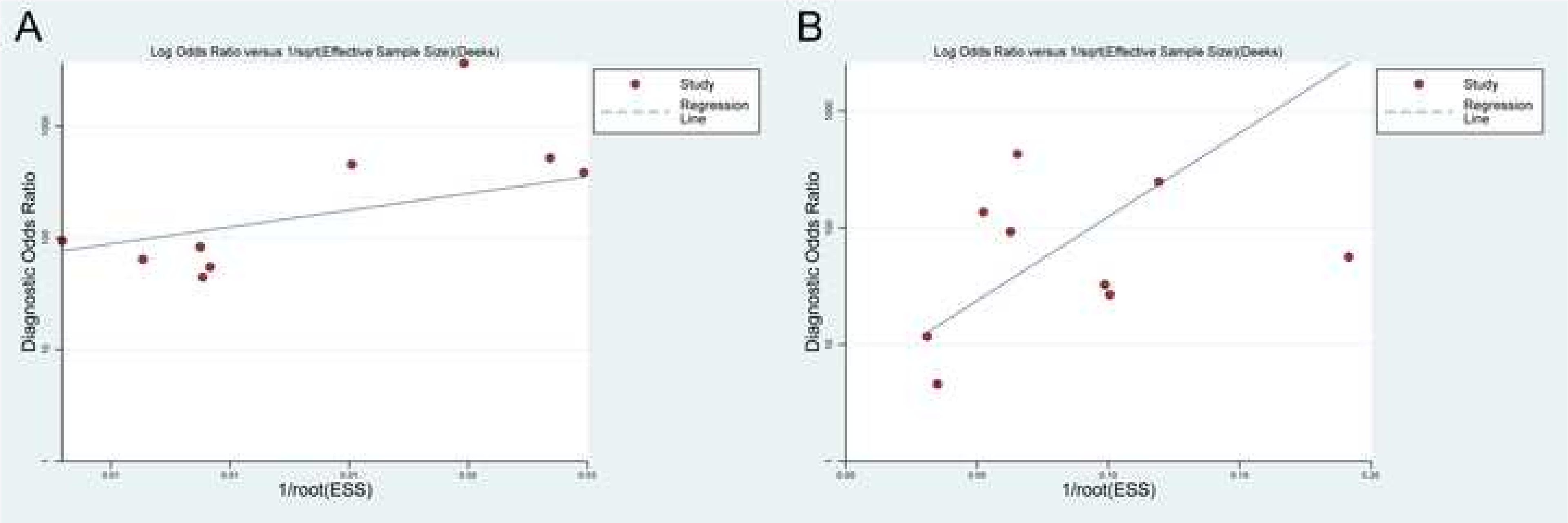

S1_Table

Click here to access/download
Supporting Information
S1_Table.doc

S2_Table

Click here to access/download
Supporting Information
S2_Table.doc

Comparison of diagnostic performance between convolutional neural networks and human endoscopists for diagnosis of colorectal polyp: a systematic review and meta-analysis

Yixin Xu[1], Yibo Wang[1], Yulin Tan[1], Cheng Xi[1], Nianyuan Ye[1], Dapeng Wu[2], Xuezhong Xu[1*]

[1] Department of General Surgery, Changzhou Wujin People's Hospital Affiliated to Jiangsu University; The Wujin Clinical College of Xuzhou Medical University, Changzhou, Jiangsu Province, China.

[2] Department of Endoscopy, Jiangsu Provincial Hospital of Traditional Chinese Medicine, Nanjing, Jiangsu, China.

***** Corresponding author

Email: xxz197001@sina.com

Abstract

Prospective randomized trials and observational studies have demonstrated that early detection, classification, and removal of neoplastic colorectal polyp (CP) can greatly improve the prevention of colorectal cancer (CRC). Currently, the effectiveness of the diagnostic performance of colonoscopy is far from satisfactory with unstable accuracy. The convolutional neural networks (CNN) system based on artificial intelligence (AI) technology has shown its potential to assist endoscopists in increasing diagnostic accuracy. However, there are still several limitations of the CNN system, and controversies exist regarding whether it can provide better diagnostic performance than human endoscopists. Therefore, the present study was conducted to address this issue. Online databases (PubMed, Web of Science, Cochrane Library, and EMBASE) were used to search for studies done up to April 2020. The quality assessment of diagnostic accuracy scale-2 (QUADAS-2) was used to evaluate the quality of the included studies. The pretest and post-test probabilities were conducted using Fagan's nomogram. Moreover, publication bias was assessed using the Deeks' funnel plot. Thirteen studies were enrolled for this meta-analysis (ranged from 2016 to 2020). In the field of CP detection, the pooled sensitivity/specificity was 90.9%/97.8%. In the present study, it was shown that the diagnostic performance of the CNN system was comparable to that of an expert but better than that of a non-expert. Meanwhile, in the field of CP classification, the pooled sensitivity and specificity was 93.5%/77.0%. The diagnostic performance of the CNN system was superior to that of the expert and non-expert. From the results

of the study, it was shown that the sensitivity, specificity, and accuracy of the CNN system were higher than that of both the experts and non-experts. Therefore, the CNN system had a satisfactory diagnostic performance for CP and could be used as a potential clinical diagnostic tool to assist endoscopists during colonoscopy.

Introduction

In 2018, it was reported that colorectal cancer (CRC) had approximately 1,800,000 new cases and 881,000 deaths, implying 1 in 10 cancer cases and deaths [1]. About 85% of CRCs were shown to have developed from precancerous polyps through genetic and epigenetic ways with a mean dwell time of at least ten years [2, 3]. Hence, early and accurate detection of colorectal polyp (CP) has great significance for the prevention of CRC. Colonoscopy is the most effective and essential method in early diagnosis and prevention of CRC through detection and removal of the neoplastic lesion before its progression to invasive cancer [4]. It was reported that at least 70% of CRCs could be prevented with regular colonoscopies and removal of polyps [4]. Therefore, it’s of great importance to achieve better diagnostic accuracy of CP for their prevention and better treatment.

Pathologically, CP can be categorized into inflammatory polyp sessile, hyperplastic polyp, serrated adenoma polyp (SSAP), and adenoma [5]. The risk of developing CRC is different for each classification. Several studies have shown that adenoma, similar to SSAP, has the highest risk for development and progression to CRC. In contrast, it is rare for hyperplastic and inflammatory polyp to develop to CRC [5, 6]. Therefore, how to accurately classify CP is important for both the endoscopists and patients, since precise differentiation of CP results in the reduction of unnecessary endoscopic resection which subsequently decreases the incidence of surgical complications, medical costs and labor burden of doctors [7].

Though, colonoscopy is effective in the early diagnosis of CRC, it is far from

being perfect and has several important limitations. Firstly, it has a relatively-high missed diagnosis rate [8]. Secondly, some neoplastic lesions are difficult to detect, even for expert endoscopists [9]. In addition, the task is time-consuming for the endoscopists and labor-intensive which can result in higher costs, especially in countries with large populations. Lastly, the diagnostic performance of colonoscopy highly depends on the working experience of endoscopists, which varies among individuals. This implies that the diagnostic accuracy of colonoscopy is unstable.

To overcome these shortcomings, several studies have reported the use of artificial intelligence to improve medical diagnosis. They are shown as follows. Recently, convolutional neural networks (CNN) have shown significant potential to assist endoscopists leading to increased diagnostic accuracy of CP during colonoscopy [10]. It is a type of the most prevalent network architectures of deep learning (DL) methods based on artificial intelligence (AI) technology. Moreover, findings from some studies showed that the CNN system could automatically classify CP, which is significantly helpful for the therapeutic decision-making process during colonoscopy [11-13]. However, this technology is still immature and far from satisfactory. Moreover, a majority of controversies exist regarding whether the CNN system can provide a better performance than the human endoscopists, and whether it is worthy of popularizing.

Therefore, the present study was conducted to compare the diagnostic performance between the CNN system and human endoscopists in the field of CP detection and classification.

Materials and Methods

Literature search strategy

A systematic literature search was conducted online for studies assessing the diagnostic value of the CNN system applied in the field of colonoscopy for colorectal polyp detection and classification. PubMed, Web of Science, Cochrane Library, and EMBASE (up to April 30, 2020) were searched with the combination of the following terms: ([“artificial intelligence”] OR [“convolutional neural networks”] OR [“deep learning”] OR [“computer-aided”]) AND ([“colonoscopy”] OR [“endoscopy”]) AND ([“colon”] OR [“rectum”] OR [“colonic”] OR [“rectal”] OR [“colorectal”]) AND ([“polyp”] OR [“polyps”]).

All article sections were carefully reviewed. Subsequently, we screened bibliographies of the retrieved articles to identify any potential source of relevant studies.

Study selection

The inclusion criteria included: (1) studies included patients with CP; (2) colonoscopy was performed to detect or classify colorectal polyps; (3) CNN system was applied to improve the diagnostic performance of colonoscopy; (4) precise diagnostic data were presented in the article; (5) if the colorectal polyps were classified, the final pathology results were provided. The exclusion criteria included: (1) the types of articles were abstracts, reviews, letters, comments, and case reports; (2) precise data were unavailable in the article; (3) animal studies and non-English

publications.

Data extraction and quality assessment

Two independent researchers (Ye and Xi) conducted the data extraction from the included studies. The information of enrolled studies included the first author's name, publication year, country, diseases concerned, training material, testing material, types of diagnostic performance, and diagnostic performance of the CNN system, expert, and non-expert. The diagnostic performance was categorized as true-positive (TP), false-positive (FP), true-negative (TN), and false-negative (FN) and was retrieved from each article. Moreover, if there was any inconsistency between the two reviewers (Ye and Xi), a discussion was conducted by a third investigator to resolve the problem.

The methodological quality and applicability of the studies included were evaluated using the quality assessment of diagnostic accuracy scale-2 (QUADAS-2) [14].

Outcomes of interests

First, pooled sensitivity, specificity, and other diagnostic indexes were calculated according to the value of TP, FP, TN, and FN, among CNN system, expert, and non-expert. Secondly, the diagnostic odds ratio (DOR) and the area (AUC) under the summary receive operating characteristic (SROC) curve, which represent overall diagnostic performance, were examined and compared among different groups. To

identify whether the differences of diagnostic performance were statistically significant, the relative diagnostic odds ratio (RDOR) was compared between each of the two groups (CNN system vs. expert; CNN system vs. non-expert; expert vs. non-expert.

Statistical analyses

Statistical analyses were performed to determine the diagnostic efficacy. The sensitivity, specificity, positive likelihood ratio (PLR), negative likelihood ratio (NLR), DOR, the AUC of SROC, and RDOR were pooled with their 95% confidence interval (CI). A diagnostic tool was considered to have strong diagnostic value, if its PLR was above 5 and NLR was below 0.2 [15]. The heterogeneity among studies was evaluated by Cochran Q and Higgins' I^2 statistics [16]. If the value of I^2 was more than 50%, and the value of P less than 0.05, indicating statistically significant heterogeneity existed, then a random-effect model was selected for pooling the data [17]. Otherwise, a fixed-effect model was utilized.

SROC was estimated based on the Moses-Littenberg method [18]. According to the AUC of SROC, the overall diagnostic performance was categorized into four levels, including reasonable (<0.75), good (0.75–0.92), very good (0.93–0.96), and excellent (≥0.97) [19].

The pretest probability was defined as the prevalence of the target condition. Meanwhile, the clinical utility of a diagnostic test was evaluated using the post-test probability, which was based on Bayes theorem [20]. They were calculated using the

likelihood ratio (LR). Post-test probability = LR × pretest probability / ([1-pretest probability] × [1-LR]) [21]. The pretest and post-test probabilities were shown in the Fagan’s nomogram.

RDOR compared between each two groups to identify statistically significant differences of diagnostic performance and was based on multivariate meta-regression analysis [22, 23]. The Deeks’ funnel plot was used to assess the publication bias.

Pooled sensitivity, specificity, accuracy, PLR, NLR, DOR, and AUC of SROC were calculated using Stata version 14.0. QUADAS-2 assessment was performed using Review Manager version 5.3, while he SROC curves were conducted using Meta-Disc version 1.4. The result was considered to be statistically significant, if *P*-value was below 0.05.

Ethical approval

As the data in this study was from previous studies, there was no need to get the ethics committee approval, follow the Declaration of Helsinki, or have patients informed consent form.

Results

Search strategy

Following the initial search through the different databases, 189 articles were identified (102 in PubMed, 31 in Web of Science, 12 in Cochrance Library, and 44

in EMBASE). Firstly, 146 duplicate studies were removed. Subsequently, the remaining 43 articles were screened after duplicates were removed. Fifteen articles, including non-English publications, reviews, abstracts, and case reports, which did not meet the inclusion criteria were rules out. Subsequently, those articles with imprecise data and irrelevant subjects were excluded after full-text articles were assessed. Finally, thirteen articles were included in the present meta-analysis (Fig 1). PRISMA flow diagram and checklist were shown in S1 and S2 Tables, respectively.

Fig 1. Flow chart of studies identified, excluded and included.

Cohort characteristics and quality of included studies

Finally, 13 studies were enrolled in this meta-analysis [10-13, 24-32]. Seven of the studies included focused on the field of CP detection, while other studies focused on the field of CP classification. In those included studies, 5 studies were performed in Japan, 4 in China, 1 in Germany, one in the USA, 1 in Norway, and 1 in Canada, respectively. All articles were published in the last four years (Table 1). Meanwhile, all studies included precise data about the diagnostic performance of the CNN system; 4 studies provided precise data about performance of expert, and 3 studies provided precise data about the performance of non-expert. Histological examination results were the golden standard in the studies done about CP classification. The diagnostic performance was categorized as TP, FP, FN, and TN (Table 2).

Table 1. Characteristics of the studies included.

**Author**	**Year**	**Country**	**type of endocytoscopes**	**type of CNN system**	**real-time use of CNN system**	**type of lesions**	**type of images**	**Training material**	**Testing material**	**Field focused**	**Testing objects**
**Lequan[27]**	2016	China	images from online database	3D fully convolutional neural networks	N/A	polyps of any size	N/A	Images	Images	Detection	CNN
**Byrne[24]**	2017	Canada	190 series colonoscopes (Olympus)	deep convolutional neural networks	Yes	diminutive polyps	NBI	Videos	Videos	Classification	CNN
**Chen[11]**	2018	China	CF-H260AZI, PCF-Q260AZI,CF-HQ290AZI (Olympus)	N/A	N/A	diminutive polyps	NBI	Images	Images	Classification	CNN/Expert/Non-expert
**Wang[10]**	2018	China	Olympus Evis Lucera CV260 (SL)/CV290 (SL) and Fujinon 4400/4450 HD	N/A	Yes	polyps of any size	N/A	Images	Images	Detection	CNN
**Renner[13]**	2018	Germany	Olympus Evis Exera III CF－HQ 190 colonoscopes	computer-assisted optical biopsy	N/A	polyps of any size	WLI/NBI	Images	Images	Classification	CNN/Expert
**Mori[12]**	2018	Japan	CFH290ECI colonoscopes (Olympus)	N/A	Yes	diminutive polyps	NBI and methylene blue staining modes	Images	Images	Classification	CNN/Expert/Non-expert
**Shin[29]**	2018	Norwa	images from	N/A	N/A	polyps of any	N/A	Images	Images	Classification	CNN

		y	online database			size					
**Urban[30]**	2018	USA	PCF-H190 colonoscopes(Olympus)	VGG16,VGG19,and ResNet50	Yes	polyps of any size	N/A	Images	Images	Detection	CNN
**Zhang[32]**	2018	China	images from online database	ResYOLO	Yes	polyps of any size	N/A	Images/	Images	Detection	CNN
**Yamada[31]**	2019	Japan	images from online database	Faster R-CNN with VGG16	Yes	polyps of any size	N/A	Images	Images	Detection	CNN
**Kudo[26]**	2019	Japan	CF-H290ECI(Olympus)	EndoBRAIN	N/A	polyps of any size	WLI/NBI	Images	WLI/NBI images	Detection	CNN/Expert/ Non-expert
**Guo[25]**	2020	Japan	Fujinon 4450 HD	YOLOv3	Yes	polyps of any size	WLI/NBI	Images	Short/full videos	Detection	CNN
**Ozawa[28]**	2020	Japan	Evis Lucera and CF Type H260AL/I, PCF Type Q260AI, Q260AZI, H290I, and H290ZI (Olympus)	Single Shot MultiBox Detector	N/A	polyps of any size	WLI/NBI	Images	Images	Classification	CNN

CNN: convolutional neural networks; NBI: Narrow band imaging; ResYOLO: residual learning modules based on YOLO; YOLO: a CNN system named you only look once; WLI: White light imaging;

Table 2. Diagnostic performance of CNN system, expert, and non-expert.

Author	Different grouping standard	CNN system				Expert				Non-expert			
		**TP**	**FP**	**TN**	**FN**	**TP**	**FP**	**TN**	**FN**	**TP**	**FP**	**TN**	**FN**
**Lequan[27]**		3062	414	9260	1251								
**Byrne[24]**		104	2	19	4								
**Chen[11]**	Group 1	181	21	75	7	183	22	74	5	183	29	67	5
	Group 2					184	33	63	4	176	33	63	12
	Group 3									154	22	74	34
	Group 4									158	11	85	30
**Wang[10]**		6404	881	20691	2345								
**Renner[13]**	Neoplastic	48	18	30	3	48	12	36	3				
	Diminutive rectosigmoid	8	6	21	0	38	9	39	14				
**Mori[12]**	Proximal-rectosigmoid	167	9	21	12	300	12	48	58	278	20	40	80
	Rectosigmoid	95	6	135	5	176	14	268	24	161	30	252	39
**Urban[30]**		7127	83	1203	228								
**Zhang[32]**		3087	398	13057	1226								
**Yamada[31]**		732	41	4094	20								
**Kudo[26]**	WLI	1260	0	700	40	603	20	330	20	920	40	460	380
	Diameter<5mm	960	0	680	40	453	20	320	47	690	236	444	310
	NBI	1260	40	660	40	608	12	338	42	807	100	600	493
	Diameter<5mm	960	40	640	40	459	12	328	41	578	97	583	422
**Guo[25]**	Short videos	2112	642	21692	1608								
	Full videos	37938	5590	78658	5672								
**Ozawa[28]**		1073	175	74	99								
	Diameter<10mm	969	171	174	84								

CNN: convolutional neural networks; FN: false-negative; FP: false-positive; NBI: narrow band imaging; TN: true negative; TP: true positive; WLI: white light imaging.

The quality of all thirteen studies included was considered moderate, according the to QUADAS-2 assessment (Fig 2). Eleven studies were considered high quality with low risk in at least five of the seven QUADAS-2 domain. For the patient selection domain, two studies introduced bias because case-control design was avoided [24, 28]. Three studies were considered to have a high concern for

applicability [11, 26, 28]. Subsequently, for the index test domain, two studies had a high risk of bias [11, 26]. Finally, there was only one study that had a high concern regarding reference standard applicability [12].

Fig 2. Methodological quality of the included 13 studies using assessment tool of QUADAS-2. (**A**) Grouped bar charts of risk of bias (left) and concerns for applicability (right). (**B**) Quality assessment for each individual study. QUADAS-2 = Quality Assessment of Diagnostic Accuracy Studies-2.

Application in the field of colorectal polyp detection

Diagnostic performance of CNN system

The results of the diagnostic performance of the CNN system are shown in Fig 3. The pooled sensitivity and specificity were 0.909 (95% CI: 0.836-0.95) and 0.978 (95% CI: 0.949-0.991), respectively. The heterpgeneity of the sensitivity (I^2=99.93, *P*=0.00) and specificity (I^2=99.87, *P*=0.00) are significant. In addition, the pooled PLR, NLR, and DOR were 42.248 (95% CI: 37.272-56.840), 0.093 (95% CI: 0.061-0.135), 208.943 (95% CI: 143.761-303.682), respectively. The AUC of SROC of the CNN system was 0.983 (95% CI: 0.971-0.992). The results are as shown in Table 3. Moreover, the PLR and NLR results of the CNN system showed that it is a good method for detecting colorectal polyps. As a result, we conducted the Fagan nomogram to evaluate the clinical application of the CNN system. The results suggested that the post-test probability of a positive result was 91% if a pretest

probability was 20%. Meanwhile, for a negative test result, a NLR of 0.09 reduced the post-test probability to 2% (Fig 4).

Fig 3. The pooled diagnostic accuracy index of CNN system in the field of CP detection. (**A**) Sensitivity, (**B**) specificity. a: short videos; b:full videos; c: WLI; d: WLI/diameter smaller than 5 mm; e: NBI; f: NBI/diameter smaller than 5mm. CNN: convolutional neural networks; CP: colorectal polyps. NBI: narrow-blue images; WLI: white-light images.

Fig 4. Fagan nomogram for CNN system in the field of CP detection. CNN: convolutional neural networks; CP: colorectal polyps.

Table 3. Diagnostic performance of CNN system, expert, and non-expert in the field of colorectal polyp detection.

Object	Sensitivity (95% CI)	Specificity (95% CI)	PLR (95% CI)	NLR (95% CI)	DOR (95% CI)	SROC (95% CI)
**CNN**	0.909 [0.836-0.954]	0.978 [0.949-0.991]	42.248 [37.272-56.840]	0.093 [0.061-0.135]	208.943 [143.761-303.682]	0.983 [0.971-0.992]
**Expert**	0.936 [0.906-0.957]	0.954 [0.941-0.964]	39.772 [24.901-46.235]	0.072 [0.046-0.099]	306.923 [179.061-526.082]	0.986 [0.952-0.983]
**Non-expert**	0.651 [0.598-0.700]	0.840 [0.734-0.909]	4.162 [2.203-7.871]	0.432 [0.352-0.524]	9.76 [4.853-19.634]	0.734 [0.712-0.790]

CNN: convolutional neural networks; DOR: diagnostic odds ratio; NLR: negative likelihood ratio; PLR: positive likelihood ratio; SROC: summary receive operating characteristic.

Diagnostic performance of expert and non-expert

As shown in Table 3, the pooled sensitivity and specificity of expert were 0.936 (95%CI: 0.906-0.957) and 0.954 (95%CI: 0.941-0.964). The heterogeneity of sensitivity is significant (I^2=85.09, P=0.00), and the heterogeneity of specificity is

moderate (I^2=30.37, *P*=0.23). Additionally, the PLR, NLR, DOR and AUC of SROC were 39.772 (95%CI: 24.901-46.235), 0.072 (95%CI: 0.046-0.099), 306.923 (95%CI: 179.061-526.082), and 0.986 (95%CI: 0.952-0.983), respectively.

Subsequently, for non-expert, the pooled sensitivity, specificity, PLR, NLR, DOR and AUC of SROC were 0.651 (95%CI: 0.598-0.700), 0.840 (95%CI: 0.734-0.909), 4.162 (95%CI: 2.203-7.871), 0.432 (95%CI: 0.352-0.524), 9.76 (95%CI: 4.853-19.634), and 0.7335 (95%CI: 0.712-0.790), respectively. The heterogeneity of sensitivity (I^2=94.42, *P*=0.00) and specificity (I^2=98.30, *P*=0.00) are significant.

Application in the field of colorectal polyp classification

Diagnostic performance of CNN system

First, the pooled sensitivity and specificity were 0.935 (95%CI: 0.915-0.947) and 0.770 (95%CI: 0.597-0.883) (Fig 5). The heterogeneity of sensitivity (I^2=96.28, *P*=0.00) and specificity (I^2=97.92, *P*=0.00) are significant. Meanwhile, the PLR, NLR, DOR, and AUC of SROC were 4.061 (95%CI: 2.155-7.652), 0.085 (95%CI: 0.060-0.120), 47.849 (95%CI: 18.638-122.842), and 0.983 (95%CI: 0.951-0.997), respectively. The Fagan nomogram suggested that the post-test probability of a positive result was 50%, if a pretest probability was 20%. Besides, for a negative test result, a NLR of 0.08 reduced the post-test probabilty to 2% (Fig 6).

Fig 5. The pooled diagnostic accuracy indexes of CNN system in the field of CP

classification. (**A**) Sensitivity, (**B**) specificity. a: neoplastic; b: diminutive rectosigmoid; c: rectosigmoid; d: proximal-rectosigmoid; CNN: convolutional neural networks; CP: colorectal polyps.

Fig 6. Fagan nomogram for CNN system in the field of CP classification. CNN: convolutional neural networks; CP: colorectal polyps.

Diagnostic performance of expert and non-expert

For the diagnostic performance of expert in the field of classification of colorectal polyps, the pooled sensitivity, specificity, PLR, NLR, DOR and AUC of SROC of expert were 0.912 (95%CI: 0.827-0.957), 0.848 (95%CI: 0.756-0.910), 6.009 (95%CI: 3.783-9.544), 0.104 (95%CI: 0.054-0.200), 57.970 (95%CI: 30.407-110.518), and 0.938 (95%CI: 0.923-0.953), respectively. The heterogeneity of sensitivity (I^2=88.17, *P*=0.00) and specificity (I^2=87.62, *P*=0.00) are significant.

Besides, the pooled sensitivity, specificity, PLR, NLR, DOR and AUC of SROC of non-expert were 0.878 (95%CI: 0.792-0.923), 0.782 (95%CI: 0.687-0.854), 3.915 (95%CI: 2.673-5.731), 0.174 (95%CI: 0.112-0.267), 25.428 (95%CI: 13.169-49.096), and 0.895 (95%CI: 0.873-0.924), respectively. The heterogeneity of sensitivity (I^2=91.38, *P*=0.00) and specificity (I^2=88.75, *P*=0.00) are significant.

All data is summarized in Table 4.

Table 4. Diagnostic performance of CNN system, expert, and non-expert in the field of colorectal polyp classification.

**Object**	**Sensitivity (95% CI)**	**Specificity (95% CI)**	**PLR (95% CI)**	**NLR (95% CI)**	**DOR (95% CI)**	**SROC (95% CI)**

**CNN**	0.935 [0.915-0.947]	0.770 [0.597-0.883]	4.061 [2.155-7.652]	0.085 [0.060-0.120]	47.849 [18.638-122.842]	0.983 [0.951-0.997]
**Expert**	0.912 [0.827-0.957]	0.848 [0.756-0.910]	6.009 [3.783-9.544]	0.104 [0.054-0.200]	57.970 [30.407-110.518]	0.938 [0.923-0.953]
**Non-expert**	0.878 [0.792-0.932]	0.782 [0.687-0.854]	3.915 [2.673-5.731]	0.174 [0.112-0.267]	25.428 [13.169-49.096]	0.895 [0.873-0.924]

CNN: convolutional neural networks; DOR: diagnostic odds ratio; NLR: negative likelihood ratio; PLR: positive likelihood ratio; SROC: summary receive operating characteristic.

The comparison of diagnostic performance among CNN system, expert, and non-expert

As shown in Fig 7, the performance of the expert was the best in the detection of CP, followed by the CNN system, while the non-expert was the least effective. However, as compared by RDOR, the difference in diagnostic performance between the expert and CNN system was statistically insignificant (Table 5).

Moreover, for CP classification, the sequence of the superiority of diagnostic performance was the CNN system, expert, and non-expert (Fig 8). However, all the differences among CNN, expert, and non-expert were statistically insignificant (Table 6).

Fig 7. Summary receiver operation characteristic (SROC) curve of diagnostic performance of CNN system, expert, and non-expert for CP detection.

Fig 8. Summary receiver operation characteristic (SROC) curve of diagnostic performance of CNN system, expert, and non-expert for CP classification.

Table 5: Comparison of diagnostic performance among CNN, expert, and non-expert in the field of colorectal polyp detection.

Object	Coefficient	Stand error	RDOR	95% CI	P
**CNN vs. Expert**	0.011	1.1780	1.01	0.08-13.17	0.9925
**CNN vs. Non-expert**	-3.531	1.1249	0.03	0.00-0.34	0.0085
**Expert vs. Non-expert**	-3.693	0.5410	0.02	0.01-0.10	0.0010

Table 6: Comparison of diagnostic performance among CNN, expert, and non-expert in the field of colorectal polyp classification.

Object	Coefficient	Stand error	RDOR	95% CI	P
**CNN vs. Expert**	-0.153	0.6669	0.86	0.21-3.59	0.8222
**CNN vs. Non-expert**	-1.0.27	0.6621	0.36	0.08-1.51	0.1467
**Expert vs. Non-expert**	-0.767	0.4904	0.46	0.16-1.37	0.1463

Publication bias and identification of sources of heterogeneity

According to Deeks' funnel plot asymmetry, there was no publication bias in pooled results of the CNN system. For CP detection, the result was $P > |t| = 0.186$. At the same time, for CP classification, the result was $P > |t| = 0.104$. They are as shown in Figs 9A and 9B. Since notable heterogeneity was observed in the pooled analysis of the CNN system in the field of CP detection and classification, meta-regression was conducted to identify the source of heterogeneity. However, no potential sources of heterogeneity were identified.

Fig 9. Deeks' funnel plot for publication bias. (**A**) CNN system for CP detection, (**B**)

CNN system for CP classification.

Discussion

In the present study, the current status of the CNN system was systematically reviewed and applied in the field of CP detection and classification. A quantitative comparison of the diagnostic value between the CNN system and human endoscopists was made. From the results of the study, the main findings were that the diagnostic performance of the CNN system was comparable to that of an expert in the field of CP detection. In contrast, the performance of the CNN system was significantly superior to that of a non-expert. Meanwhile, in the field of CP classification, the diagnostic performance of the CNN system was better than either expert or non-expert, despite the differences were statistically insignificant.

The American Society of Gastrointestinal Endoscopy published Preservation and Incorporation of Valuable Endoscopic Innovations (PIVI) statement in 2015 to address the resect and discard strategy [33]. This strategy set the threshold of a diagnose-and-leave strategy for small colorectal polyps as NPV≥90%. At the same time, the threshold of a resect-and-discard strategy is above 90% of the agreement with histopathology for post-polypectomy surveillance intervals[34]. These set standards are very high and hard to achieve, even for experienced endoscopists. Besides, the task of endoscopists is time-consuming as well as labor-intensive. Some studies have shown that endoscopic detections and predictions led to a rather low diagnostic accuracy rate, especially in the case of non-expert use [35, 36].

Hence, the need for the application and use of technological support. This is because these studies have shown that computer-aided diagnosis of endoscopic images using AI has the potential to surpass the diagnostic accuracy of trained specialist. AI may also provide more accurate results without interobserver differences, especially between experts and non-experts.

Currently, a considerable number of studies have been done focusing on the development of the CNN system that assisted human endoscopists. In the field of colonoscopy, its function is mainly divided into two categories: detection and classification. For CP detection, we found that the DOR of the CNN system was 208.943 [95%CI: 143.761-303.682], which is lower than that of expert (306.923 [95%CI: 173.061-526.082]), but significantly better than that of non-expert (9.76 [95%CI: 4.853-19.634]). Similar results which indicated that an experienced expert had better diagnostic performance than the CNN system were also found in the field of *Helicobacter pylori* (HP) infection status evaluation [37]. However, it is important to consider that not every endoscopist has working experience for decades to qualify being an expert. Most of the endoscopists are ordinary physicians, trainees, and novices. Therefore, the application of the CNN system can significantly increase their diagnostic accuracy. Moreover, some studies showed that these non-expert endoscopists achieved a better diagnostic performance during endoscopy after the AI training course [38, 39]. Hence, the AI technologies have a potential to be applid not only as a clinical ancillary diagnostic tool, but also as an endoscopist training method.

Moreover, it would be highly beneficial if endoscopic observation can distinguish neoplastic CP from hyperplastic CP. This is because the removal of lesions without malignant potential is not only expensive, but also leads to high post-procedure complications [40]. Thus, the precise classification of CP will significantly improve the cost-effectiveness of colonoscopy. However, the task of precisely classifying the different types of CP is rather difficult. For instance, lesions with indistinct borders, flat and depressed features in conventional adenomas are challenging to distinguish from surrounding normal mucosa. This is especially common when the bowel preparation is inadequate or the mucosa is capped by mucus or intestinal residue [9]. A study conducted by Kuiper et al. showed that the sensitivity/specificity of classification of diminutive CP was only 77.0%/78.8%, which was far from satisfactory [41]. In this study, it was also shown that the sensitivity/specificity of a non-expert in the field of CP classification was 87.8%/78.2%. Thus, the benefits of optical CP classification may remain limited to experts. However, as earlier mentioned, not every endoscopist is an expert. Hence, the emergence of AI technology has greatly overcome this limitation. In the present study, we found that the diagnostic performance of the CNN system was better than both the expert and non-expert, despite the difference between the CNN system and expert being statistically insignificant. However, due to the complexity of classification technology, the DOR of the CNN system applied in the field of CP classification (47.849 [95%CI: 18.638-122.842]) was weaker than that in the field of CP detection (208.943 [95%CI: 143.761-303.682]). A similar CNN-DL system was

also used for diagnosis and classification of proximal gastric precancerous conditions, including chronic atrophic gastritis, intestinal metaplasia, and dysplasia [42]. This system achieved a sensitivity of 93.5%, a specificity of 94.0%, and an accuracy of 93.5%, which were much better than both the less and more experienced endoscopists.

However, CNN technology has several limitations.

First, most of the images and videos extracted for CNN system training are highly qualified, which usually leads to selection bias. These systems are often unable to distinguish lesions from low-quality materials. Their diagnostic performance is excellent in the training set but weak in the clinical practice.

Secondly, images and videos of rare lesions are challenging to find, such as subtle flat colonic lesions and morphology types. They are insufficient in either hospitals' independent or online databases, which will result in insufficient training of the CNN system. This will further lead to high misdiagnostic rates of infrequent diseases.

Finally, most studies included in the present review trained their CNN systems with stationary images or image frames extracted from colonoscopic videos. It may restrict the ability of real-time implementation of the CNN system. Moreover, due to the lack of calculating power of computer processors and the complexity of technical processes, the latency of the decision-making process of most systems is not satisfactory, which will subsequently disturb the endoscopist during colonoscopy. The ability to work in real-time during endoscopy should be incorporated.

With the advancements of AI technology, it can be predicted that an ideal CNN system will be developed to overcome these limitations. It may precisely distinguish different lesions from normal surrounding mucosa, including those rare lesions. Meanwhile, it may assist endoscopists simultaneously during endoscopy with almost undetectable latency. Even more, it may provide the type, location, size, depth, and other relevant information of lesions.

In the present meta-analysis study, there are some limitations to this study that should be acknowledged here. First, studies focused on this field are limited since the CNN system applied in the field of endoscopy has recently emerged and is not yet prevalent. Secondly, the sample size of comparison between the CNN system and human endoscopists is small, which may lead to selection bias. Thirdly, although there is no publication bias in the present study, since letters, reviews, as well as articles not published in English were excluded, selective reporting bias may exist. Fourthly, although meta-regression analysis was performed to identify the potential sources of heterogeneity, due to the limitation of the sample size and the variables collected from studies included, the exploration of heterogeneity may still be inadequate. Finally, a majority of studies included were retrospective and used different types of training and testing materials, which is assumed to have a potential bias.

Conclusions

In conclusion, our systematic review and meta-analysis suggested that the CNN

system achieved comparable diagnostic performance to that of an expert, but better performance than that of a non-expert, in the field of CP detection. Moreover, in the field of CP classification, the CNN system demonstrated better diagnostic performance than the human endoscopists regardless of the level of working experience. Although there are still some limitations of the CNN system, it can be popularized in clinical practice with relative-high diagnostic accuracy and thus enhance the diagnostic performance of endoscopists.

Acknowledgements

The authors thank Dr. Peng Jiang and Dr. Hai-Feng Tang for their critical reading and informative advice during the study process. Meanwhile, the authors thank Freescience for language polishing. There was no financial support received.

Author contributions

Conceptualization: Yixin Xu.

Data curation: Yixin Xu, Yulin Tan,.

Formal analysis: Dapeng Wu, Cheng Xi.

Methodology: Cheng Xi.

Software: Nianyuan Ye.

Writing – original draft: Yixin Xu, Yibo Wang.

Writing – review and editing: Xuezhong Xu.

Conflict of interest:

None of the authors have any financial interests related to this study to disclose.

Reference

1. Bray F, Ferlay J, Soerjomataram I, Siegel RL, Torre LA, Jemal A. Global cancer statistics 2018: GLOBOCAN estimates of incidence and mortality worldwide for 36 cancers in 185 countries. CA Cancer J Clin. 2018;68(6):394-424. doi: 10.3322/caac.21492. PubMed PMID: 30207593.
2. Kuntz KM, Lansdorp-Vogelaar I, Rutter CM, Knudsen AB, van Ballegooijen M, Savarino JE, et al. A systematic comparison of microsimulation models of colorectal cancer: the role of assumptions about adenoma progression. Med Decis Making. 2011;31(4):530-9. doi: 10.1177/0272989X11408730. PubMed PMID: 21673186.
3. Strum WB. Colorectal Adenomas. N Engl J Med. 2016;375(4):389-90. doi: 10.1056/NEJMc1604867. PubMed PMID: 27464213.
4. Winawer SJ, Zauber AG, Ho MN, O'Brien MJ, Gottlieb LS, Sternberg SS, et al. Prevention of colorectal cancer by colonoscopic polypectomy. The National Polyp Study Workgroup. N Engl J Med. 1993;329(27):1977-81. PubMed PMID: 8247072.
5. Ijspeert JEG, Bastiaansen BAJ, van Leerdam ME, Meijer GA, van Eeden S, Sanduleanu S, et al. Development and validation of the WASP classification system for optical diagnosis of adenomas, hyperplastic polyps and sessile serrated adenomas/polyps. Gut. 2016;65(6):963-70. doi: 10.1136/gutjnl-2014-308411. PubMed PMID: 25753029.
6. Ijspeert JEG, Bevan R, Senore C, Kaminski MF, Kuipers EJ, Mroz A, et al. Detection rate of serrated polyps and serrated polyposis syndrome in colorectal cancer screening cohorts: a European overview. Gut. 2017;66(7):1225-32. doi: 10.1136/gutjnl-2015-310784. PubMed PMID: 26911398.
7. Hassan C, Pickhardt PJ, Rex DK. A resect and discard strategy would improve cost-effectiveness of colorectal cancer screening. Clinical gastroenterology and hepatology : the official clinical practice journal of the American Gastroenterological Association. 2010;8(10). doi: 10.1016/j.cgh.2010.05.018. PubMed PMID: 20621680.
8. van Rijn JC, Reitsma JB, Stoker J, Bossuyt PM, van Deventer SJ, Dekker E. Polyp miss rate determined by tandem colonoscopy: a systematic review. Am J Gastroenterol. 2006;101(2):343-50. PubMed PMID: 16454841.
9. Yamada M, Sakamoto T, Otake Y, Nakajima T, Kuchiba A, Taniguchi H, et al. Investigating endoscopic features of sessile serrated adenomas/polyps by using narrow-band imaging with optical magnification. Gastrointest Endosc. 2015;82(1):108-17. doi: 10.1016/j.gie.2014.12.037. PubMed PMID: 25840928.
10. Wang P, Xiao X, Glissen Brown JR, Berzin TM, Tu M, Xiong F, et al. Development and validation of a deep-learning algorithm for the detection of polyps during

colonoscopy. Nat Biomed Eng. 2018;2(10):741-8. doi: 10.1038/s41551-018-0301-3. PubMed PMID: 31015647.

11. Chen PJ, Lin MC, Lai MJ, Lin JC, Lu HH, Tseng VS. Accurate Classification of Diminutive Colorectal Polyps Using Computer-Aided Analysis. Gastroenterology. 2018;154(3):568-75. doi: 10.1053/j.gastro.2017.10.010. PubMed PMID: 29042219.

12. Mori Y, Kudo SE, Misawa M, Saito Y, Ikematsu H, Hotta K, et al. Real-Time Use of Artificial Intelligence in Identification of Diminutive Polyps During Colonoscopy: A Prospective Study. Ann Intern Med. 2018;169(6):357-66. doi: 10.7326/M18-0249. PubMed PMID: 30105375.

13. Renner J, Phlipsen H, Haller B, Navarro-Avila F, Saint-Hill-Febles Y, Mateus D, et al. Optical classification of neoplastic colorectal polyps - a computer-assisted approach (the COACH study). Scand J Gastroenterol. 2018;53(9):1100-6. doi: 10.1080/00365521.2018.1501092. PubMed PMID: 30270677.

14. Whiting PF, Rutjes AWS, Westwood ME, Mallett S, Deeks JJ, Reitsma JB, et al. QUADAS-2: a revised tool for the quality assessment of diagnostic accuracy studies. Ann Intern Med. 2011;155(8):529-36. doi: 10.7326/0003-4819-155-8-201110180-00009. PubMed PMID: 22007046.

15. Deeks JJ. Systematic reviews in health care: Systematic reviews of evaluations of diagnostic and screening tests. BMJ. 2001;323(7305):157-62. PubMed PMID: 11463691.

16. Higgins JPT, Thompson SG, Deeks JJ, Altman DG. Measuring inconsistency in meta-analyses. BMJ. 2003;327(7414):557-60. PubMed PMID: 12958120.

17. Jackson D, White IR, Thompson SG. Extending DerSimonian and Laird's methodology to perform multivariate random effects meta-analyses. Stat Med. 2010;29(12):1282-97. doi: 10.1002/sim.3602. PubMed PMID: 19408255.

18. Moses LE, Shapiro D, Littenberg B. Combining independent studies of a diagnostic test into a summary ROC curve: data-analytic approaches and some additional considerations. Stat Med. 1993;12(14):1293-316. PubMed PMID: 8210827.

19. Jones CM, Athanasiou T. Summary receiver operating characteristic curve analysis techniques in the evaluation of diagnostic tests. Ann Thorac Surg. 2005;79(1):16-20. PubMed PMID: 15620907.

20. Jaeschke R, Guyatt GH, Sackett DL. Users' guides to the medical literature. III. How to use an article about a diagnostic test. B. What are the results and will they help me in caring for my patients? The Evidence-Based Medicine Working Group. JAMA. 1994;271(9):703-7. PubMed PMID: 8309035.

21. Jaeschke R, Guyatt G, Sackett DL. Users' guides to the medical literature. III. How to use an article about a diagnostic test. A. Are the results of the study valid? Evidence-Based Medicine Working Group. JAMA. 1994;271(5):389-91. PubMed PMID: 8283589.

22. Jackson D, Riley R, White IR. Multivariate meta-analysis: potential and promise. Stat Med. 2011;30(20):2481-98. doi: 10.1002/sim.4172. PubMed PMID: 21268052.

23. Ian RW. Multivariate random-effects meta-regression: Updates to mvmeta. Stata Journal. 2011;11(2):255-70. doi: 10.1177/1536867X1101100206.

24. Byrne MF, Chapados N, Soudan F, Oertel C, Linares Pérez M, Kelly R, et al.

Real-time differentiation of adenomatous and hyperplastic diminutive colorectal polyps during analysis of unaltered videos of standard colonoscopy using a deep learning model. Gut. 2019;68(1). doi: 10.1136/gutjnl-2017-314547. PubMed PMID: 29066576.
25. Guo Z, Nemoto D, Zhu X, Li Q, Aizawa M, Utano K, et al. A polyp detection algorithm can detect small polyps: An ex vivo reading test compared with endoscopists. Dig Endosc. 2020. doi: 10.1111/den.13670. PubMed PMID: 32173917.
26. Kudo SE, Misawa M, Mori Y, Hotta K, Ohtsuka K, Ikematsu H, et al. Artificial Intelligence-assisted System Improves Endoscopic Identification of Colorectal Neoplasms. Clin Gastroenterol Hepatol. 2019. doi: 10.1016/j.cgh.2019.09.009. PubMed PMID: 31525512.
27. Lequan Y, Hao C, Qi D, Jing Q, Pheng Ann H. Integrating Online and Offline Three-Dimensional Deep Learning for Automated Polyp Detection in Colonoscopy Videos. IEEE J Biomed Health Inform. 2017;21(1):65-75. doi: 10.1109/JBHI.2016.2637004. PubMed PMID: 28114049.
28. Ozawa T, Ishihara S, Fujishiro M, Kumagai Y, Shichijo S, Tada T. Automated endoscopic detection and classification of colorectal polyps using convolutional neural networks. Therap Adv Gastroenterol. 2020;13:1756284820910659. doi: 10.1177/1756284820910659. PubMed PMID: 32231710; PubMed Central PMCID: PMCPMC7092386.
29. Shin Y, Balasingham I. Automatic polyp frame screening using patch based combined feature and dictionary learning. Comput Med Imaging Graph. 2018;69:33-42. doi: 10.1016/j.compmedimag.2018.08.001. PubMed PMID: 30172091.
30. Urban G, Tripathi P, Alkayali T, Mittal M, Jalali F, Karnes W, et al. Deep Learning Localizes and Identifies Polyps in Real Time With 96% Accuracy in Screening Colonoscopy. Gastroenterology. 2018;155(4):1069-78 e8. doi: 10.1053/j.gastro.2018.06.037. PubMed PMID: 29928897; PubMed Central PMCID: PMCPMC6174102.
31. Yamada M, Saito Y, Imaoka H, Saiko M, Yamada S, Kondo H, et al. Development of a real-time endoscopic image diagnosis support system using deep learning technology in colonoscopy. Sci Rep. 2019;9(1):14465. doi: 10.1038/s41598-019-50567-5. PubMed PMID: 31594962; PubMed Central PMCID: PMCPMC6783454.
32. Zhang R, Zheng Y, Poon CCY, Shen D, Lau JYW. Polyp detection during colonoscopy using a regression-based convolutional neural network with a tracker. Pattern Recognit. 2018;83:209-19. doi: 10.1016/j.patcog.2018.05.026. PubMed PMID: 31105338; PubMed Central PMCID: PMCPMC6519928.
33. Chandrasekhara V, Desilets D, Falk GW, Inoue H, Romanelli JR, Savides TJ, et al. The American Society for Gastrointestinal Endoscopy PIVI (Preservation and Incorporation of Valuable Endoscopic Innovations) on peroral endoscopic myotomy. Gastrointest Endosc. 2015;81(5). doi: 10.1016/j.gie.2014.12.007. PubMed PMID: 25799295.

34. Rex DK, Kahi C, O'Brien M, Levin TR, Pohl H, Rastogi A, et al. The American Society for Gastrointestinal Endoscopy PIVI (Preservation and Incorporation of Valuable Endoscopic Innovations) on real-time endoscopic assessment of the histology of diminutive colorectal polyps. Gastrointest Endosc. 2011;73(3):419-22. doi: 10.1016/j.gie.2011.01.023. PubMed PMID: 21353837.
35. Rees CJ, Rajasekhar PT, Wilson A, Close H, Rutter MD, Saunders BP, et al. Narrow band imaging optical diagnosis of small colorectal polyps in routine clinical practice: the Detect Inspect Characterise Resect and Discard 2 (DISCARD 2) study. Gut. 2017;66(5):887-95. doi: 10.1136/gutjnl-2015-310584. PubMed PMID: 27196576.
36. Schachschal G, Mayr M, Treszl A, Balzer K, Wegscheider K, Aschenbeck J, et al. Endoscopic versus histological characterisation of polyps during screening colonoscopy. Gut. 2014;63(3):458-65. doi: 10.1136/gutjnl-2013-304562. PubMed PMID: 23812324.
37. Shichijo S, Endo Y, Aoyama K, Takeuchi Y, Ozawa T, Takiyama H, et al. Application of convolutional neural networks for evaluating Helicobacter pylori infection status on the basis of endoscopic images. Scandinavian journal of gastroenterology. 2019;54(2):158-63. doi: 10.1080/00365521.2019.1577486. PubMed PMID: 30879352.
38. Sehgal V, Rosenfeld A, Graham DG, Lipman G, Bisschops R, Ragunath K, et al. Machine Learning Creates a Simple Endoscopic Classification System that Improves Dysplasia Detection in Barrett's Oesophagus amongst Non-expert Endoscopists. Gastroenterol Res Pract. 2018;2018:1872437. doi: 10.1155/2018/1872437. PubMed PMID: 30245711.
39. Cai S-L, Li B, Tan W-M, Niu X-J, Yu H-H, Yao L-Q, et al. Using a deep learning system in endoscopy for screening of early esophageal squamous cell carcinoma (with video). Gastrointest Endosc. 2019;90(5). doi: 10.1016/j.gie.2019.06.044. PubMed PMID: 31302091.
40. Abu Dayyeh BK, Thosani N, Konda V, Wallace MB, Rex DK, Chauhan SS, et al. ASGE Technology Committee systematic review and meta-analysis assessing the ASGE PIVI thresholds for adopting real-time endoscopic assessment of the histology of diminutive colorectal polyps. Gastrointest Endosc. 2015;81(3). doi: 10.1016/j.gie.2014.12.022. PubMed PMID: 25597420.
41. Kuiper T, Marsman WA, Jansen JM, van Soest EJ, Haan YCL, Bakker GJ, et al. Accuracy for optical diagnosis of small colorectal polyps in nonacademic settings. Clinical gastroenterology and hepatology : the official clinical practice journal of the American Gastroenterological Association. 2012;10(9). doi: 10.1016/j.cgh.2012.05.004. PubMed PMID: 22609999.
42. Guimarães P, Keller A, Fehlmann T, Lammert F, Casper M. Deep-learning based detection of gastric precancerous conditions. Gut. 2020;69(1):4-6. doi: 10.1136/gutjnl-2019-319347. PubMed PMID: 31375599.

Supporting information

S1 Table. PRISMA flow diagram.

S2 Table. PRISMA checklist.

Dear Editor:

Thank you and the reviewers for your valuable suggestions. We have carefully read through the comments and made proper revisions. Our responses to the reviewer's questions are listed below. We greatly appreciated your time and efforts to improve our manuscript for publication.

Sincerely,

Yixin Xu

Journal Requirements:
When submitting your revision, we need you to address these additional requirements.

1. Please ensure that your manuscript meets PLOS ONE's style requirements, including those for file naming. The PLOS ONE style templates can be found at https://journals.plos.org/plosone/s/file?id=wjVg/PLOSOne_formatting_sample_main_body.pdf and https://journals.plos.org/plosone/s/file?id=ba62/PLOSOne_formatting_sample_title_authors_affiliations.pdf

Response:

We have revised the manuscript following the style requirements.

2. Please include the date(s) on which you accessed the databases or records to obtain the data used in your study.

Response:

We completed the data collection on April 30, 2020. It was mentioned on Page 6, Line 117.

3. Thank you for quantifying study heterogeneity.
Please provide more detailed reporting on your results, for example, by reporting your Q or I^2 statistics.

Response:

In the field of CP detection, we add the value of I^2 and *P* for CNN system on Page 14, Line 248-249; for expert on Page 15-16, Line 274-276; for non-expert on Page 16, Line 282-284. In the field of CP classification, we add the value of I^2 and *P* for CNN system on Page 16, Line 289-290; for expert on Page 17, Line 309-310; for non-expert on Page 17, Line 314-315. All these data added were marked in green.

4. We note that you state in your manuscript "The design and protocol of the present study were approved by our institutional review board." Since this is a systematic review and does not include non-public data, this statement is not necessary and may be taken out.
If you would like to keep this statement, please provide the full name of your ethics committee and the ethics approval number.

Response:

We have taken out this statement "The design and protocol of the present study were approved by our institutional review board.", and added "As the data in this study was from previous studies, there was no need to get the ethics committee approval, follow the Declaration of Helsinki, or have patients informed consent form." to the Ethical approval part.

5. PLOS requires an ORCID iD for the corresponding author in Editorial Manager on papers submitted after December 6th, 2016. Please ensure that you have an ORCID iD and that it is validated in Editorial Manager. To do this, go to 'Update my Information' (in the upper left-hand corner of the main menu), and click on the Fetch/Validate link next to the ORCID field. This will take you to the ORCID site and allow you to create a new iD or authenticate a pre-existing iD in Editorial Manager. Please see the following video for instructions on linking an ORCID iD to your Editorial Manager account: https://www.youtube.com/watch?v=_xcclfuvtxQ

Response:

We have added the ORCID to the Editorial Manager.

6. Please include captions for your Supporting Information files at the end of your manuscript, and update any in-text citations to match accordingly. Please see our Supporting Information guidelines for more information: http://journals.plos.org/plosone/s/supporting-information

Response:

We have added the supporting information at the end of the manuscript and cited on Page 10, Line 206.

Reviewer 1#

This is an interesting systematic review article written by Xu et al. This review is well-written and described using appropriate statistical methods. The authors conducted meta-analysis to compare the diagnostic performance of artificial intelligence (AI) for detecting and classifying the colorectal polyp to the human endoscopists. This study must be of interest to the reader and will provide useful knowledge to the reader. I have one comment for the authors.

1. Shouldn't the classification of polyps be divided into normal observation, magnification (80×) and super magnification (520×, microscopic observation) for analysis? Please revisit and consider this point.

Response:

Thanks a lot for your summary and kind remarks.

To your constructive suggestion, we have carefully reviewed all the references in our meta-analysis. We found that only few studies included in our analysis mentioned the magnification type of their images used to train the CNN system. Only Kudo et al. and Mori

et al. used the super magnification (520×). As a result, we did not divide the classification into different groups. Besides, we found that the classification system of CNN applied in colonoscopy mainly focused on determining whether the polyps, especially those diminutive polyps, were hyperplastic or neoplastic. For neoplastic polyps, simple resection may be not sufficient. Endoscopic Mucosal Resection (EMR), Endoscopic submucosal dissection (ESD) or even surgery is the optimal curative option. The process of classification of polyps were finished using the routine colonoscopy images (some systems using narrow-band images). Besides, magnification colonoscopy is currently not commercially available worldwide.

Reviewer 2#

The meta-analysis presented by Dr Xu and colleagues addresses an interesting topic, that is the performance of convolutional neural networks for detection and classification of colorectal polyps.

The fact that computer-aided colonoscopy and artificial intelligence are hot-topics is demonstrated by the very recent publication of other dedicated systematic reviews and meta-analysis, such as Barua et al, Endoscopy 2020 or Hassan et al, GIE 2020 (detection) and Lui et al, GIE 2020 (detection and classification).

In this meta-analysis, the authors only focus of CNNs, a specific type of artificial intelligence.

Unfortunately, the paper presented by dr. Xu has some important limitations:

Response:

Thank you for this summary of our paper.

I started my manuscript on January 10, 2020. Before that, I have not found any similar article through Searching PubMed and other database. I thought this subject about artificial intelligence applied in the field of colonoscopy was interesting and “hot”. I finished my manuscript on June, and submitted it to *PLOS ONE* on July 22. I have not realized that there were three articles about this subject have already been published. Those limitations you mentioned were addressed below.

1. The search strategy seems well designed, and I appreciated the effort to analyze the quality of included studies and the possibility of publication bias. However, at least one recent full-study about CNNs that met the inclusion criteria was not included in the analysis (Urban 2018, Gastroenterology); also some abstracts (such as Misawa 2019 GIE, Matsui 2019 GIE, LUI 2019 GIE) were not included. The choice whether to include or not abstracts can be discussed (even if in a meta-analysis they are usually included), however abstracts were not included in the exclusion criteria.

Response:

Urban 2018, Gastroenterology was already included in my analysis. I marked it in red in Table 1 and Table 2. It is also the number 30 in my reference list (in red).

We have carefully read these abstracts (Misawa 2019 GIE, Matsui 2019 GIE, LUI 2019 GIE) and we found that they could not provide precise data for analysis. As a result, we decided not to include them. For more accurate expression, we added "abstracts" to the exclusion criteria part and marked it in red (Page 6, Line 132).

2. Data extraction from the selected studies was not so rigorous and clear. For example, apparently for reference 13 (Renner et al) authors have included the performances of CNN considering either high-confidence predictions or "standard" predictions (see table 2), with potential duplication of data and overestimation of performance.

Response:

Thank you for this constructive suggestion. We carefully reviewed the article of Renner et al., and found maybe the high-confidence predictions had the potential of duplication of data. As a result, we decided to delete the data of high-confidence, and recalculated the whole part of classification. There are so many changes, and all of them were marked in red. (Page 16, Line 288, 289, 291, 292, 294, 295; Page 17, Line 307-309; Page 19, Line 346). Table 4, Table 6,

Fig. 5, Fig. 6, Fig. 8, and Fig. 9 were remade. Besides, we added some notes to Fig. 3 (Page 15, Line 261-264), and Fig. 5 (Page 17, Line 298-299). All these changes were marked in red.

3. Moreover, for reference 25 (Guo et al) the authors considered per-frame sensitivity and specificity of short and long videos; however, in order to provide clinically useful information (similar to polyp or adenoma detection rates) per-video sensitivity and specificity (reported in table 3 of the cited paper for the 100 short videos) should have been used instead. In table 3 of the paper by Guo et al are also reported the diagnostic performances of 2 experts e 2 non-experts physicians that were not included in the metanalysis.

Response:

There are two reasons that we did use per-frame sensitivity and specificity instead of per-video sensitivity and specificity. First, almost of the articles included in our analysis used colonoscopy images instead of videos to compare the diagnostic performance between the CNN system and human endoscopists. In order to ensure the consistency of the whole analysis, we chose the per-frame sensitivity and specificity. Second, for per-video sensitivity and specificity, Guo et al. did not provide enough data for us to analyze. In table 3 of their paper, they only provide the data of per-video sensitivity, but not the data of per-video specificity. As a result, we cannot get the data of (TP, TN, FP, and FN).

When we read the paper of Guo et al., we have found that they reported the diagnostic performance of experts and non-experts. But the data of human endoscopists are per-video, not per-frame. Based on the reasons we mentioned above, we decided to use the data of per-frame instead of per-video. So we did not include the data of human endoscopists.

4. Similarly, for Wang et al (ref 31) the 1633 polyps (and not the total number of polyps images) should have been considered.

Response:

The total 1633 polyps belong to Dataset A and C.

In the paper of Wang et al., they used four databases to validate the diagnostic performance of CNN system. Dataset A and B contained colonoscopy images. Dataset C and D contained colonoscopy video. However, they only provide precise data of Dataset A. As a result, we chose included the data of Dataset A. When we search for the references, we have found almost all authors used colonoscopy images to compare diagnostic performance between CNN system and human endoscopists. So we decided to choose colonoscopy images as the main source of our analysis.

5. I think that the use of the Fagan Nomogram to calculate post-test probability considering positive and negative likelihood ratios is not really indicated in this field.

Response:

The Fangan Nomogram is based on Bayes' Theorem. It is often used to evaluate the clinical application of a diagnostic method or tool. The pre-test probability is calculated using Bayes' Theorem. The post-test probability of LR-positive means the probability of patients suspected of this disease would get a positive diagnostic result using this diagnostic method or tool. While the post-test probability of LR-negative means the probability of patients who are not suspected of this disease would suffer it after this diagnostic method or tool gives them a negative diagnostic result.

After carefully thinking about the meaning of Fangan Nomogram, we think the application of it in the field of detection and classification of colorectal polyps may be appropriate.

6. The differential performance according to dimension of polyps (diminutive and non-diminutive polyps) should have been analysed

Response:

Sometimes colorectal polyps are hard to detect and classify, especially for the diminutive ones. So maybe the diagnostic performance would be different between diminutive polyps and

non-diminutive polyps. After carefully reviewed all the references, we found that seven studies provided data about diminutive polyps (Chen et al., Renner et al., Mori et al., Ozawa et al., Shin et al., Byrne et al., and Kudo et al.). Among them, Kudo et al. focused on the detection of Polyps, and Chen et al., Mori et al., and Byrne et al. mainly focused on diminutive polyps. Because only one study provided data about diminutive polyps in the detection part, so we thought it was not necessary to perform a subgroup analysis. In the subgroup analysis of diminutive polyps in the classification part, the result showed the performance of the CNN and expert had just slightly changed. Probably, the reason is because only two studies (Renner et al., and Ozawa et al.) provided data about non-diminutive polyps. After careful consideration, we decided not to add the subgroup analysis to the manuscript, due to the slight change of the results and the consistency of the article.

The subgroup analysis was shown below:

**Object**	**Sensitivity (95% CI)**	**Specificity (95% CI)**	**PLR (95% CI)**	**NLR (95% CI)**	**DOR (95% CI)**	**SROC (95% CI)**
**CNN**	0.935 [0.915-0.947]	0.770 [0.597-0.883]	4.061 [2.155-7.652]	0.085 [0.060-0.120]	47.849 [18.638-122.842]	0.983 [0.951-0.997]
**Expert**	0.912 [0.827-0.957]	0.848 [0.756-0.910]	6.009 [3.783-9.544]	0.104 [0.054-0.200]	57.970 [30.407-110.518]	0.938 [0.923-0.953]
**Non-expert**	0.878 [0.792-0.932]	0.782 [0.687-0.854]	3.915 [2.673-5.731]	0.174 [0.112-0.267]	25.428 [13.169-49.096]	0.895 [0.873-0.924]
**Diminutive polyps**						
**CNN**	0.948 [0.925-0.965]	0.812 [0.673-0.900]	5.037 [2.741-9.258]	0.064 [0.040-0.101]	79.210 [29.347-213.791]	0.965 [0.941-0.972]
**Expert**	0.903 [0.808-0.954]	0.864 [0.777-0.921]	4.659 [3.099-8.818]	0.112 [0.057-0.221]	49.389 [30.057-117.346]	0.921 [0.901-0.965]
**Non-expert**	0.878 [0.792-0.932]	0.782 [0.687-0.854]	3.915 [2.673-5.731]	0.174 [0.112-0.267]	25.428 [13.169-49.096]	0.895 [0.873-0.924]

Note: The sample size of non-diminutive polyps is too small, so it cannot be analyzed. The data of non-expert in whole group are identical to those in diminutive polyps group, so the results are just the same.

7. The paper requires a deep linguistic revision (grammatical mistakes, repetitions…), possibly by an English mother-tongue revisor.

Response:

We have already sent this paper to language polishing. (All changes were marked in blue.)

Other observations:

8. Reference 10 does not refer to detection of colonic lesions, but of early esophageal squamous cell carcinoma/preneoplastic lesions in the esophagus. The sentence in the introduction related to this reference should be revised.

Response:

We are sorry for this mistake. We have revised this mistake and cited another reference.

9. Both table 1 and 2 should contain the reference numbers of the included papers in order to make the tables easier to read. Moreover, I suggest to list the papers according to the year of publication.

Response:

We added the reference number to Table 1 and 2 and listed papers according to year of publication. Besides, I fixed two references' names and marked them in red. (Kuo is altered to Kudo; Yu is altered to Lequan)

10. Table 1 should also include more details about the imaging modalities included in the different studies. For example, the use of dedicated endocytoscopes in the studies referred as 12 and 25 should be reported. Also information about the use of real-time analysis by the CNN systems, and the type of lesions included (only diminutive polyps or polyps of any size) in the different studies should be highlighted in the table.

Response:

We have added information of type of endocytoscopes, type of CNN system, real-time use of CNN system, type of lesions, and type of images into Table 1, and marked them in red.

11. In the paper it is stated that 7 of the included studies focused on detection; however in table 1 only 6 papers have detection been reported as field focus; similarly, 5 studies should include experts human endoscopists but only four papers are reported in table 1 and 2.

Response:

Actually, there are 7 studies focused on detection, they are Lequan et al., Wang et al., Urban et al., Zhang et al., Yamada et al., Kudo et al., and Guo et al.. In fact, we made a mistake. There are only 4 studies provided data about experts and 3 studies provided data about non-experts. We revised it and marked it in red. (Page 10, Line 216)

12. The paper by Yu (2016), included in table 1 and 2 is not reported in the references.

Response:

Yu (2016) is actually Lequan (2016), we just mistook his/her last name. We have revised it and marked it in red.

Review 3#

1. There are some spelling mistakes:

- line 186, page 15: Results instead of resules

- line 211, page 17, table 2: proximal rectosigmoid instead of poximal rectosigmoid

- line 360, page 25: ordinary physicians instead of phyisicians

Response:

We have revised all the spelling mistakes, and already sent the manuscript to language polishing.

2. There are missing the technical explanations of the different CNN-systems you compared in your analysis: Why are some CNN-systems better for CP detection and others for CP classification? Is it justified to compare different CNN-systems that provide different features? Do they have different characteristics concerning deep learning?

Response:

For CP detection training, authors chose images contained CP and those did not contain CP. Their purpose is to train the CNN system to distinguish CP from normal colorectal mucosa. This task is also very difficult. Because flat, small and isochromatic polyps are associated with a high miss rate, even for experts.

While for CP classification training, authors used images contained hyperplastic or neoplastic CP. The authors used NBI or magnification images to train CNN system to classify different pathological type of CP. For hyperplastic CP, the treatment is just simple resection. While for neoplastic CP, it is not enough. Endoscopic Mucosal Resection (EMR), Endoscopic submucosal dissection (ESD) or even surgery is the optimal curative option. If the accuracy of classification function of CNN system is high enough, endoscopists can trust the diagnostic results provided by it and make a simultaneous therapeutic decision while colonoscopy.

Maybe the details of algorithms of detection and classification are slightly different. But rare authors provided information about these differences. However, they are all based on CNN basically.

3. There is also missing the description of the different classification-systems of CP used by different authors in different studies.

Response:

We have added information of different type of CNN system used in different studies in Table 1.
