# Supplementary material for: Comparison of diagnostic performance between convolutional neural networks and human endoscopists for diagnosis of colorectal polyp: A systematic review and meta-analysis
Source: PLoS One. 2021 Feb 16;16(2):e0246892. doi: 10.1371/journal.pone.0246892 (PMC7886136; doi:10.1371/journal.pone.0246892)
Supplement: Attachment — Submitted filename: Response to reviewers.docx [file pone.0246892.s004.docx]

Dear Editor:

Thank you and the reviewers for your valuable suggestions. We have carefully read through the comments and made proper revisions. Our responses to the reviewer’s questions are listed below. We greatly appreciated your time and efforts to improve our manuscript for publication.

Sincerely,

Yixin Xu

**Journal Requirements:**

**When submitting your revision, we need you to address these additional requirements.**

**1. Please ensure that your manuscript meets PLOS ONE's style requirements, including those for file naming. The PLOS ONE style templates can be found at**

**https://journals.plos.org/plosone/s/file?id=wjVg/PLOSOne_formatting_sample_main_body.pdf and**

**https://journals.plos.org/plosone/s/file?id=ba62/PLOSOne_formatting_sample_title_authors_affiliations.pdf**

Response:

We have revised the manuscript following the style requirements.

**2. Please include the date(s) on which you accessed the databases or records to obtain the data used in your study.**

Response:

We completed the data collection on April 30, 2020. It was mentioned on Page 6, Line 117.

**3. Thank you for quantifying study heterogeneity.**

**Please provide more detailed reporting on your results, for example, by reporting your Q or I^2 statistics.**

Response:

In the field of CP detection, we add the value of I^2^ and *P* for CNN system on Page 14, Line 248-249; for expert on Page 15-16, Line 274-276; for non-expert on Page 16, Line 282-284.

In the field of CP classification, we add the value of I^2^ and *P* for CNN system on Page 16, Line 289-290; for expert on Page 17, Line 309-310; for non-expert on Page 17, Line 314-315.

All these data added were marked in green.

**4. We note that you state in your manuscript "The design and protocol of the present study were approved by our institutional review board."**

**Since this is a systematic review and does not include non-public data, this statement is not necessary and may be taken out.**

**If you would like to keep this statement, please provide the full name of your ethics committee and the ethics approval number.**

Response:

We have taken out this statement “The design and protocol of the present study were approved by our institutional review board.”, and added “As the data in this study was from previous studies, there was no need to get the ethics committee approval, follow the Declaration of Helsinki, or have patients informed consent form.” to the Ethical approval part.

**5. PLOS requires an ORCID iD for the corresponding author in Editorial Manager on papers submitted after December 6th, 2016. Please ensure that you have an ORCID iD and that it is validated in Editorial Manager. To do this, go to ‘Update my Information’ (in the upper left-hand corner of the main menu), and click on the Fetch/Validate link next to the ORCID field. This will take you to the ORCID site and allow you to create a new iD or authenticate a pre-existing iD in Editorial Manager. Please see the following video for instructions on linking an ORCID iD to your Editorial Manager account: https://www.youtube.com/watch?v=_xcclfuvtxQ**

Response:

We have added the ORCID to the Editorial Manager.

**6. Please include captions for your Supporting Information files at the end of your manuscript, and update any in-text citations to match accordingly. Please see our Supporting Information guidelines for more information:** [**http://journals.plos.org/plosone/s/supporting-information**](http://about:blank/)

Response:

We have added the supporting information at the end of the manuscript and cited on Page 10, Line 206.

**Reviewer 1#**

**This is an interesting systematic review article written by Xu et al. This review is well-written and described using appropriate statistical methods. The authors conducted meta-analysis to compare the diagnostic performance of artificial intelligence (AI) for detecting and classifying the colorectal polyp to the human endoscopists. This study must be of interest to the reader and will provide useful knowledge to the reader. I have one comment for the authors.**

**1. Shouldn't the classification of polyps be divided into normal observation, magnification (80×) and super magnification (520×, microscopic observation) for analysis? Please revisit and consider this point.**

Response:

Thanks a lot for your summary and kind remarks.

To your constructive suggestion, we have carefully reviewed all the references in our meta-analysis. We found that only few studies included in our analysis mentioned the magnification type of their images used to train the CNN system. Only Kudo et al. and Mori et al. used the super magnification (520**×**). As a result, we did not divide the classification into different groups. Besides, we found that the classification system of CNN applied in colonoscopy mainly focused on determining whether the polyps, especially those diminutive polyps, were hyperplastic or neoplastic. For neoplastic polyps, simple resection may be not sufficient. Endoscopic Mucosal Resection (EMR), Endoscopic submucosal dissection (ESD) or even surgery is the optimal curative option. The process of classification of polyps were finished using the routine colonoscopy images (some systems using narrow-band images). Besides, magnification colonoscopy is currently not commercially available worldwide.

**Reviewer 2#**

**The meta-analysis presented by Dr Xu and colleagues addresses an interesting topic, that is the performance of convolutional neural networks for detection and classification of colorectal polyps.
The fact that computer-aided colonoscopy and artificial intelligence are hot-topics is demonstrated by the very recent publication of other dedicated systematic reviews and meta-analysis, such as Barua et al, Endoscopy 2020 or Hassan et al, GIE 2020 (detection) and Lui et al, GIE 2020 (detection and classification).
In this meta-analysis, the authors only focus of CNNs, a specific type of artificial intelligence.
Unfortunately, the paper presented by dr. Xu has some important limitations:**

Response:

Thank you for this summary of our paper.

I started my manuscript on January 10, 2020. Before that, I have not found any similar article through Searching PubMed and other database. I thought this subject about artificial intelligence applied in the field of colonoscopy was interesting and “hot”. I finished my manuscript on June, and submitted it to *PLOS ONE* on July 22. I have not realized that there were three articles about this subject have already been published. Those limitations you mentioned were addressed below.

**1. The search strategy seems well designed, and I appreciated the effort to analyze the quality of included studies and the possibility of publication bias. However, at least one recent full-study about CNNs that met the inclusion criteria was not included in the analysis (Urban 2018, Gastroenterology); also some abstracts (such as Misawa 2019 GIE, Matsui 2019 GIE, LUI 2019 GIE) were not included. The choice whether to include or not abstracts can be discussed (even if in a meta-analysis they are usually included), however abstracts were not included in the exclusion criteria.**

Response:

Urban 2018, Gastroenterology was already included in my analysis. I marked it in red in Table 1 and Table 2. It is also the number 30 in my reference list (in red).

We have carefully read these abstracts (Misawa 2019 GIE, Matsui 2019 GIE, LUI 2019 GIE) and we found that they could not provide precise data for analysis. As a result, we decided not to include them. For more accurate expression, we added “abstracts” to the exclusion criteria part and marked it in red (Page 6, Line 132).

**2. Data extraction from the selected studies was not so rigorous and clear.
For example, apparently for reference 13 (Renner et al) authors have included the performances of CNN considering either high-confidence predictions or "standard" predictions (see table 2), with potential duplication of data and overestimation of performance.**

Response:

Thank you for this constructive suggestion. We carefully reviewed the article of Renner et al., and found maybe the high-confidence predictions had the potential of duplication of data. As a result, we decided to delete the data of high-confidence, and recalculated the whole part of classification. There are so many changes, and all of them were marked in red. (Page 16, Line 288, 289, 291, 292, 294, 295; Page 17, Line 307-309; Page 19, Line 346). Table 4, Table 6, Fig. 5, Fig. 6, Fig. 8, and Fig. 9 were remade. Besides, we added some notes to Fig. 3 (Page 15, Line 261-264), and Fig. 5 (Page 17, Line 298-299). All these changes were marked in red.

**3. Moreover, for reference 25 (Guo et al) the authors considered per-frame sensitivity and specificity of short and long videos; however, in order to provide clinically useful information (similar to polyp or adenoma detection rates) per-video sensitivity and specificity (reported in table 3 of the cited paper for the 100 short videos) should have been used instead. In table 3 of the paper by Guo et al are also reported the diagnostic performances of 2 experts e 2 non-experts physicians that were not included in the metanalysis.**

Response:

There are two reasons that we did use per-frame sensitivity and specificity instead of per-video sensitivity and specificity. First, almost of the articles included in our analysis used colonoscopy images instead of videos to compare the diagnostic performance between the CNN system and human endoscopists. In order to ensure the consistency of the whole analysis, we chose the per-frame sensitivity and specificity. Second, for per-video sensitivity and specificity, Guo et al. did not provide enough data for us to analyze. In table 3 of their paper, they only provide the data of per-video sensitivity, but not the data of per-video specificity. As a result, we cannot get the data of (TP, TN, FP, and FN).

When we read the paper of Guo et al., we have found that they reported the diagnostic performance of experts and non-experts. But the data of human endoscopists are per-video, not per-frame. Based on the reasons we mentioned above, we decided to use the data of per-frame instead of per-video. So we did not include the data of human endoscopists.

**4. Similarly, for Wang et al (ref 31) the 1633 polyps (and not the total number of polyps images) should have been considered.**

Response:

The total 1633 polyps belong to Dataset A and C.

In the paper of Wang et al., they used four databases to validate the diagnostic performance of CNN system. Dataset A and B contained colonoscopy images. Dataset C and D contained colonoscopy video. However, they only provide precise data of Dataset A. As a result, we chose included the data of Dataset A. When we search for the references, we have found almost all authors used colonoscopy images to compare diagnostic performance between CNN system and human endoscopists. So we decided to choose colonoscopy images as the main source of our analysis.

**5. I think that the use of the Fagan Nomogram to calculate post-test probability considering positive and negative likelihood ratios is not really indicated in this field.**

Response:

The Fangan Nomogram is based on Bayes’ Theorem. It is often used to evaluate the clinical application of a diagnostic method or tool. The pre-test probability is calculated using Bayes’ Theorem. The post-test probability of LR-positive means the probability of patients suspected of this disease would get a positive diagnostic result using this diagnostic method or tool. While the post-test probability of LR-negative means the probability of patients who are not suspected of this disease would suffer it after this diagnostic method or tool gives them a negative diagnostic result.

After carefully thinking about the meaning of Fangan Nomogram, we think the application of it in the field of detection and classification of colorectal polyps may be appropriate.

**6. The differential performance according to dimension of polyps (diminutive and non-diminutive polyps) should have been analysed**

Response:

Sometimes colorectal polyps are hard to detect and classify, especially for the diminutive ones. So maybe the diagnostic performance would be different between diminutive polyps and non-diminutive polyps. After carefully reviewed all the references, we found that seven studies provided data about diminutive polyps (Chen et al., Renner et al., Mori et al., Ozawa et al., Shin et al., Byrne et al., and Kudo et al.). Among them, Kudo et al. focused on the detection of Polyps, and Chen et al., Mori et al., and Byrne et al. mainly focused on diminutive polyps. Because only one study provided data about diminutive polyps in the detection part, so we thought it was not necessary to perform a subgroup analysis. In the subgroup analysis of diminutive polyps in the classification part, the result showed the performance of the CNN and expert had just slightly changed. Probably, the reason is because only two studies (Renner et al., and Ozawa et al.) provided data about non-diminutive polyps. After careful consideration, we decided not to add the subgroup analysis to the manuscript, due to the slight change of the results and the consistency of the article.

The subgroup analysis was shown below:

| **Object** | **Sensitivity (95% CI)** | **Specificity**  **(95% CI)** | **PLR**  **(95% CI)** | **NLR**  **(95% CI)** | **DOR**  **(95% CI)** | **SROC**  **(95% CI)** |
| --- | --- | --- | --- | --- | --- | --- |
| **CNN** | 0.935 [ 0.915-0.947] | 0.770 [0.597-0.883] | 4.061 [2.155-7.652] | 0.085 [0.060-0.120] | 47.849 [ 18.638-122.842] | 0.983 [ 0.951-0.997] |
| **Expert** | 0.912 [0.827-0.957] | 0.848 [0.756-0.910] | 6.009 [3.783-9.544] | 0.104 [0.054-0.200] | 57.970 [ 30.407-110.518] | 0.938 [0.923-0.953] |
| **Non-expert** | 0.878 [0.792-0.932] | 0.782 [0.687-0.854] | 3.915 [2.673-5.731] | 0.174 [0.112-0.267] | 25.428 [13.169-49.096] | 0.895 [0.873-0.924] |
| **Diminutive polyps** |  |  |  |  |  |  |
| **CNN** | 0.948 [0.925-0.965] | 0.812 [0.673-0.900] | 5.037 [2.741-9.258] | 0.064 [0.040-0.101] | 79.210 [29.347-213.791] | 0.965 [0.941-0.972] |
| **Expert** | 0.903 [0.808-0.954] | 0.864 [0.777-0.921] | 4.659 [3.099-8.818] | 0.112 [0.057-0.221] | 49.389 [30.057-117.346] | 0.921 [0.901-0.965] |
| **Non-expert** | 0.878 [0.792-0.932] | 0.782 [0.687-0.854] | 3.915 [2.673-5.731] | 0.174 [0.112-0.267] | 25.428 [13.169-49.096] | 0.895 [0.873-0.924] |

Note: The sample size of non-diminutive polyps is too small, so it cannot be analyzed. The data of non-expert in whole group are identical to those in diminutive polyps group, so the results are just the same.

**7. The paper requires a deep linguistic revision (grammatical mistakes, repetitions…), possibly by an English mother-tongue revisor.**

Response:

We have already sent this paper to language polishing. (All changes were marked in blue.)

**Other observations:
8. Reference 10 does not refer to detection of colonic lesions, but of early esophageal squamous cell carcinoma/preneoplastic lesions in the esophagus. The sentence in the introduction related to this reference should be revised.**

Response:

We are sorry for this mistake. We have revised this mistake and cited another reference.

**9. Both table 1 and 2 should contain the reference numbers of the included papers in order to make the tables easier to read. Moreover, I suggest to list the papers according to the year of publication.**

Response:

We added the reference number to Table 1 and 2 and listed papers according to year of publication. Besides, I fixed two references’ names and marked them in red. (Kuo is altered to Kudo; Yu is altered to Lequan )

**10. Table 1 should also include more details about the imaging modalities included in the different studies. For example, the use of dedicated endocytoscopes in the studies referred as 12 and 25 should be reported. Also information about the use of real-time analysis by the CNN systems, and the type of lesions included (only diminutive polyps or polyps of any size) in the different studies should be highlighted in the table.**

Response:

We have added information of type of endocytoscopes, type of CNN system, real-time use of CNN system, type of lesions, and type of images into Table 1, and marked them in red.

**11. In the paper it is stated that 7 of the included studies focused on detection; however in table 1 only 6 papers have detection been reported as field focus; similarly, 5 studies should include experts human endoscopists but only four papers are reported in table 1 and 2.**

Response:

Actually, there are 7 studies focused on detection, they are Lequan et al., Wang et al., Urban et al., Zhang et al., Yamada et al., Kudo et al., and Guo et al.. In fact, we made a mistake. There are only 4 studies provided data about experts and 3 studies provided data about non-experts. We revised it and marked it in red. (Page 10, Line 216)

**12. The paper by Yu (2016), included in table 1 and 2 is not reported in the references.**

Response:

Yu (2016) is actually Lequan (2016), we just mistook his/her last name. We have revised it and marked it in red.

**Review 3#**

**1. There are some spelling mistakes:
- line 186, page 15: Results instead of resules
- line 211, page 17, table 2: proximal rectosigmoid instead of poximal rectosigmoid
- line 360, page 25: ordinary physicians instead of phyisicians**

Response:

We have revised all the spelling mistakes, and already sent the manuscript to language polishing.

**2. There are missing the technical explanations of the different CNN-systems you compared in your analysis: Why are some CNN-systems better for CP detection and others for CP classification? Is it justified to compare different CNN-systems that provide different features? Do they have different characteristics concerning deep learning?**

Response:

For CP detection training, authors chose images contained CP and those did not contain CP. Their purpose is to train the CNN system to distinguish CP from normal colorectal mucosa. This task is also very difficult. Because flat, small and isochromatic polyps are associated with a high miss rate, even for experts.

While for CP classification training, authors used images contained hyperplastic or neoplastic CP. The authors used NBI or magnification images to train CNN system to classify different pathological type of CP. For hyperplastic CP, the treatment is just simple resection. While for neoplastic CP, it is not enough. Endoscopic Mucosal Resection (EMR), Endoscopic submucosal dissection (ESD) or even surgery is the optimal curative option. If the accuracy of classification function of CNN system is high enough, endoscopists can trust the diagnostic results provided by it and make a simultaneous therapeutic decision while colonoscopy.

Maybe the details of algorithms of detection and classification are slightly different. But rare authors provided information about these differences. However, they are all based on CNN basically.

**3. There is also missing the description of the different classification-systems of CP used by different authors in different studies.**

Response:

We have added information of different type of CNN system used in different studies in Table 1.
