# Supplementary material for: Comparison of diagnostic performance between convolutional neural networks and human endoscopists for diagnosis of colorectal polyp: A systematic review and meta-analysis
Source: PLoS One. 2021 Feb 16;16(2):e0246892. doi: 10.1371/journal.pone.0246892 (PMC7886136; doi:10.1371/journal.pone.0246892)
Supplement: Attachment — Submitted filename: Re-Review PLOS one CNNs polyps.docx [file pone.0246892.s005.docx]

I would like to thank the authors for the changes apported to their paper according to my and other reviewers’ comments. I think the quality of the paper has globally improved with these changes.

However, I still have doubts about the methodology adopted for the extraction of data. I think that some choices (such as the use of per-video or per-frame performances, or the use of high-confidence vs low-confidence predictions) are arbitrary and therefore not fully compatible with a meta-analysis. I think that a correctly conducted meta-analysis should start from a clear protocol in which all these questions are addressed in advance; at least authors should try to discuss and justify these choices in the paper (methods section).

My specific comments to the changes apported to the paper are listed below the answers provided by the authors.

Thank you for this summary of our paper.

I started my manuscript on January 10, 2020. Before that, I have not found any similar

article through Searching PubMed and other database. I thought this subject about

artificial intelligence applied in the field of colonoscopy was interesting and “hot”. I

finished my manuscript on June, and submitted it to PLOS ONE on July 22. I have not

realized that there were three articles about this subject have already been published.

Those limitations you mentioned were addressed below.

1. The search strategy seems well designed, and I appreciated the effort to analyze the

quality of included studies and the possibility of publication bias. However, at least one

recent full-study about CNNs that met the inclusion criteria was not included in the

analysis (Urban 2018, Gastroenterology); also some abstracts (such as Misawa 2019

GIE, Matsui 2019 GIE, LUI 2019 GIE) were not included. The choice whether to

include or not abstracts can be discussed (even if in a meta-analysis they are usually

included), however abstracts were not included in the exclusion criteria.

Response:

Urban 2018, Gastroenterology was already included in my analysis. I marked it in red

in Table 1 and Table 2. It is also the number 30 in my reference list (in red).

We have carefully read these abstracts (Misawa 2019 GIE, Matsui 2019 GIE, LUI 2019

GIE) and we found that they could not provide precise data for analysis. As a result, we

decided not to include them. For more accurate expression, we added “abstracts” to

the exclusion criteria part and marked it in red (Page 6, Line 132).

1. **Response: It may be reasonable not to include abstracts in a meta-analysis. Thank you for having clarified this choice.**

2. Data extraction from the selected studies was not so rigorous and clear.

For example, apparently for reference 13 (Renner et al) authors have included the

performances of CNN considering either high-confidence predictions or "standard"

predictions (see table 2), with potential duplication of data and overestimation of

performance.

Response:

Thank you for this constructive suggestion. We carefully reviewed the article of Renner

et al., and found maybe the high-confidence predictions had the potential of duplication

of data. As a result, we decided to delete the data of high-confidence, and recalculated

the whole part of classification. There are so many changes, and all of them were

marked in red. (Page 16, Line 288, 289, 291, 292, 294, 295; Page 17, Line 307-309;

Page 19, Line 346). Table 4, Table 6, Fig. 5, Fig. 6, Fig. 8, and Fig. 9 were remade.

Besides, we added some notes to Fig. 3 (Page 15, Line 261-264), and Fig. 5 (Page 17,

Line 298-299). All these changes were marked in red.

2. **Response: thank you for this major change. I think that this choice, such as the others subsequently listed, should somehow be discussed and motivated in the Methods section of the study.**

3. Moreover, for reference 25 (Guo et al) the authors considered per-frame sensitivity

and specificity of short and long videos; however, in order to provide clinically useful

information (similar to polyp or adenoma detection rates) per-video sensitivity and

specificity (reported in table 3 of the cited paper for the 100 short videos) should have

been used instead. In table 3 of the paper by Guo et al are also reported the diagnostic

performances of 2 experts e 2 non-experts physicians that were not included in the

metanalysis.

Response:

There are two reasons that we did use per-frame sensitivity and specificity instead of

per-video sensitivity and specificity. First, almost of the articles included in our analysis

used colonoscopy images instead of videos to compare the diagnostic performance

between the CNN system and human endoscopists. In order to ensure the consistency

of the whole analysis, we chose the per-frame sensitivity and specificity. Second, for

per-video sensitivity and specificity, Guo et al. did not provide enough data for us to

analyze. In table 3 of their paper, they only provide the data of per-video sensitivity, but

not the data of per-video specificity. As a result, we cannot get the data of (TP, TN, FP,

and FN).

When we read the paper of Guo et al., we have found that they reported the diagnostic

performance of experts and non-experts. But the data of human endoscopists are per-

video, not per-frame. Based on the reasons we mentioned above, we decided to use

the data of per-frame instead of per-video. So we did not include the data of human

endoscopists.

4. Similarly, for Wang et al (ref 31) the 1633 polyps (and not the total number of polyps

images) should have been considered.

Response:

The total 1633 polyps belong to Dataset A and C.

In the paper of Wang et al., they used four databases to validate the diagnostic

performance of CNN system. Dataset A and B contained colonoscopy images. Dataset

C and D contained colonoscopy video. However, they only provide precise data of

Dataset A. As a result, we chose included the data of Dataset A. When we search for

the references, we have found almost all authors used colonoscopy images to

compare diagnostic performance between CNN system and human endoscopists. So

we decided to choose colonoscopy images as the main source of our analysis.

3 **And 4. Thank you for these comments. Please try to discuss these choices in the method section.**

5. I think that the use of the Fagan Nomogram to calculate post-test probability

considering positive and negative likelihood ratios is not really indicated in this field.

Response:

The Fangan Nomogram is based on Bayes’ Theorem. It is often used to evaluate the

clinical application of a diagnostic method or tool. The pre-test probability is calculated

using Bayes’ Theorem. The post-test probability of LR-positive means the probability of

patients suspected of this disease would get a positive diagnostic result using this

diagnostic method or tool. While the post-test probability of LR-negative means the

probability of patients who are not suspected of this disease would suffer it after this

diagnostic method or tool gives them a negative diagnostic result.

After carefully thinking about the meaning of Fangan Nomogram, we think the

application of it in the field of detection and classification of colorectal polyps may be

appropriate.

5. **Of course I am aware of the meaning of the Fagan Nomogram; my doubts refer to the clinical, real life application of the Bayes’ theorem in this field. In particular, I criticize the arbitrary choice of the 20% pre-test probability of a patient having a polyp or of a polyp being an adenoma chosen in the paper.
   I find not clear the sentence “the pretest probability was defined as the prevalence of the target condition” (line 175, statistical analysis): The pre-test probability of a patient having a polyp of course will change accordingly to the patient’s characteristics and to indication for colonoscopy; the pre-test probability of a polyp being an adenoma depends from the characteristics of the polyp and is even more difficult to estimate in terms of %.
   If you want to keep the Fagan nomogram in the paper, I suggest choosing more appropriate pre-test probabilities (for example, expected polyp or adenoma detection rates in screening population derived from previous studies). However, I am really skeptical about the use of Fagan nomogram especially in the field of polyps’ classification: what does it mean that a polyp has 20% pre-test probability of being an adenoma? I think that an endoscopist generally has a much clearer idea of the nature of a polyp.**

6. The differential performance according to dimension of polyps (diminutive and non-

diminutive polyps) should have been analysed

Response:

Sometimes colorectal polyps are hard to detect and classify, especially for the

diminutive ones. So maybe the diagnostic performance would be different between

diminutive polyps and non-diminutive polyps. After carefully reviewed all the

references, we found that seven studies provided data about diminutive polyps (Chen

et al., Renner et al., Mori et al., Ozawa et al., Shin et al., Byrne et al., and Kudo et al.).

Among them, Kudo et al. focused on the detection of Polyps, and Chen et al., Mori et

al., and Byrne et al. mainly focused on diminutive polyps. Because only one study

provided data about diminutive polyps in the detection part, so we thought it was not

necessary to perform a subgroup analysis. In the subgroup analysis of diminutive

polyps in the classification part, the result showed the performance of the CNN and

expert had just slightly changed. Probably, the reason is because only two studies

(Renner et al., and Ozawa et al.) provided data about non-diminutive polyps. After

careful consideration, we decided not to add the subgroup analysis to the manuscript,

due to the slight change of the results and the consistency of the article.

The subgroup analysis was shown below:

ObjectSensitivity (95% CI)Specificity

(95% CI)PLR

(95% CI)NLR

(95% CI)DOR

(95% CI)SROC

(95% CI)

CNN 0.935 [ 0.915-0.947]0.770 [0.597-0.883]4.061 [2.155-7.652]0.085 [0.060-

0.120]47.849 [ 18.638-122.842]0.983 [ 0.951-0.997]

Expert0.912 [0.827-0.957]0.848 [0.756-0.910]6.009 [3.783-9.544]0.104 [0.054-

0.200]57.970 [ 30.407-110.518]0.938 [0.923-0.953]

Non-expert0.878 [0.792-0.932]0.782 [0.687-0.854]3.915 [2.673-5.731]0.174 [0.112-

0.267]25.428 [13.169-49.096]0.895 [0.873-0.924]

Diminutive polyps

CNN0.948 [0.925-0.965]0.812 [0.673-0.900]5.037 [2.741-9.258]0.064 [0.040-

0.101]79.210 [29.347-213.791]0.965 [0.941-0.972]

Expert0.903 [0.808-0.954]0.864 [0.777-0.921]4.659 [3.099-8.818]0.112 [0.057-

0.221]49.389 [30.057-117.346]0.921 [0.901-0.965]

Non-expert0.878 [0.792-0.932]0.782 [0.687-0.854]3.915 [2.673-5.731]0.174 [0.112-

0.267]25.428 [13.169-49.096]0.895 [0.873-0.924]

Note: The sample size of non-diminutive polyps is too small, so it cannot be analyzed.

The data of non-expert in whole group are identical to those in diminutive polyps group,

so the results are just the same.

**6. Thank you for the effort done to perform this subgroup analysis. Maybe you could consider inserting this subgroup analysis in the paper as a supplementary data, briefly discussing the results in the main paper.**

7. The paper requires a deep linguistic revision (grammatical mistakes, repetitions...),

possibly by an English mother-tongue revisor.

Response:

We have already sent this paper to language polishing. (All changes were marked in

blue.)
**7. The paper has for sure improved. However, I still find that some sections could be slightly modified in order to avoid repetitions (for example, lines 40-41 and 43-44 in the abstract; lines 205-206 and 210 in the results) and ease reading (line 447 in the discussion section). Some minor spelling mistakes are still present: line 187, he SROC (instead of the SROC); line 248, heterpgeneity; line 395, applid;**

Other observations:

8. Reference 10 does not refer to detection of colonic lesions, but of early esophageal

squamous cell carcinoma/preneoplastic lesions in the esophagus. The sentence in the

introduction related to this reference should be revised.

Response:

We are sorry for this mistake. We have revised this mistake and cited another

reference.
**8. ok**

9. Both table 1 and 2 should contain the reference numbers of the included papers in

order to make the tables easier to read. Moreover, I suggest to list the papers

according to the year of publication.

Response:

We added the reference number to Table 1 and 2 and listed papers according to year

of publication. Besides, I fixed two references’ names and marked them in red. (Kuo is

altered to Kudo; Yu is altered to Lequan )

10. Table 1 should also include more details about the imaging modalities included in

the different studies. For example, the use of dedicated endocytoscopes in the studies

referred as 12 and 25 should be reported. Also information about the use of real-time

analysis by the CNN systems, and the type of lesions included (only diminutive polyps

or polyps of any size) in the different studies should be highlighted in the table.

Response:

We have added information of type of endocytoscopes, type of CNN system, real-time

use of CNN system, type of lesions, and type of images into Table 1, and marked them

in red.
**9 and 10. Thanks to the changes done, tables 1 and 2 are easier to read now and more informative. I only suggest to modify “type of endocytoscopes” in table 1 with “type of endoscopes”, since endocytoscopes were used only in few papers**.

11. In the paper it is stated that 7 of the included studies focused on detection;

however in table 1 only 6 papers have detection been reported as field focus; similarly,

5 studies should include experts human endoscopists but only four papers are reported

in table 1 and 2.

Response:

Actually, there are 7 studies focused on detection, they are Lequan et al., Wang et al.,

Urban et al., Zhang et al., Yamada et al., Kudo et al., and Guo et al.. In fact, we made

a mistake. There are only 4 studies provided data about experts and 3 studies

provided data about non-experts. We revised it and marked it in red. (Page 10, Line

216)
**11. ok**

12. The paper by Yu (2016), included in table 1 and 2 is not reported in the references.

Response:

Yu (2016) is actually Lequan (2016), we just mistook his/her last name. We have

revised it and marked it in red.
**12. Ok**

**Other comments:**

- **For the study by Kudo et al (reference 26), I also think that considering both White light images and NBI images may lead to duplication of data, because they refer to the same polyps.**
- **In the abstract it is stated that “the diagnostic performance of the CNN system was superior to that of the expert and non-expert” (lines 43-44). However, the meta-analysis did not find any statistically significant difference. Modify the sentence accordingly.**
- **I suggest to split the paragraph “The comparison of diagnostic performance among CNN system, expert and non-expert” (page 18), by positioning the comparisons in the fields of detection and classification immediately after the relative paragraphes “diagnostic performance of expert and non-expert”.**
