# Supplementary material for: Comparison of diagnostic performance between convolutional neural networks and human endoscopists for diagnosis of colorectal polyp: A systematic review and meta-analysis
Source: PLoS One. 2021 Feb 16;16(2):e0246892. doi: 10.1371/journal.pone.0246892 (PMC7886136; doi:10.1371/journal.pone.0246892)
Supplement: Attachment — Submitted filename: response to reviewers.docx [file pone.0246892.s007.docx]

Dear Pro. Ping He:

Thank you and the reviewers for all your valuable suggestions. We have carefully read through the comments and made proper revisions. Our responses to the reviewer’s questions are listed below. We greatly appreciated your time and efforts to improve our manuscript for publication.

Sincerely,

Xuezhong Xu

Reviewer 2#

**1. I carefully analyzed the results showed in table 2. In this table are reported the diagnostic categories (TP, FP, TN, FN) of all the studies included in the meta-analysis. I tried to calculate by myself sensitivity for detection and I found different values from the ones reported in the paper. For example, considering only the seven papers labeled as “detection” studies in table 1, I calculated for the CNN system a sensitivity (that is TP/TP+FN) of 64902/77412=0.838, that is different from the reported 0.909.**

Response: We recalculated the whole analysis. We do the analysis using the midas package of STATA. We found the result of pooled sensitivity or specificity was not simply calculated as TP/(TP+FN). The data must be transformed somehow according to the heterogeneity. As a result, maybe the sensitivity or specificity you calculated was not right.

**2. After careful review, I have noticed that the study by Kudo et al [17] has been erroneously classified as detection study in table 1, while it is a classification study. Even excluding this study, however, sensitivity for CNN for detection is different (60462/77252= 0.782) from the reported one.**

Response: We modified Kudo to the field of CP classification and do all the analysis again.

**3. I have also another doubt: were all the data included in table 2 used to calculate diagnostic performances? If yes, there was a duplication of small lesions in Kudo [17] (reported both all the lesions, that include small lesions, and separately only lesions < 5 mm), Ozawa [28] (all lesions and only lesions < 10 mm) and Renner [15] (all lesions and diminutive rectosigmoid).**

Response: We deleted all the data which had the potential risk of duplication. Initially, we wanted to perform a subgroup analysis for diminutive polyps, but STATA could not analyze data with sample size smaller than 4.

**4. I suggest to split table 2 in two different tables, one including only detection studies and one classification studies. This will make easier for readers (and for reviewers) to independently control the diagnostic performances reported in the paper and will allow to clarify the reasons for the difference I found in detection sensitivity.**

Response: We split Table 2 into two different tables.

**5. Modify the reported “field” of the study by Kudo [17] in table 1 (detection  classification). I suggest to carefully review all the papers included in order to correctly classify them into detection and classification studies.**

Response: We modified Kudo to the field of CP classification and do all the analysis again.

**6. I suggest to carefully review again all the data inserted (for example, I noticed that for Renner et al. are reported 99 results in table 2, but the original paper includes 100 polyps) and to recalculate all the reported diagnostic performances.**

Response: We checked all data included again and modified the data of Renner et al.

**7. Were small lesions in Kudo, Ozawa and Renner considered twice as they were reported in table 2? If yes, this is a mistake that has to be corrected (duplication of small polyps)**

Response: We deleted all the data which had the potential risk of duplication. Initially, we wanted to perform a subgroup analysis for diminutive polyps, but STATA could not analyze data with sample size smaller than 4.

**8. One more limitation of the study is that more than half of the data come from a single study (Guo et al [16], especially because of the inclusion of “full-videos” data. The results for detection are, for this reason, strongly “guided” by this study.**

**Can you provide, in addition to the general analysis, another analysis for detection excluding the large study by Guo et al? It could be very informative and add strength to the meta-analysis results. You should also briefly discuss this aspect in the discussion.**

Response: We did subgroup analysis without the data of Guo et al. and discussed it in Line 392-396, Page 20.

**9. Minor concerns:**

**Minor concerns:**

**- Lines 27-28: I find too severe the sentence “significantly unsatisfactory” to describe the efficacy of colonoscopy.**

**- Line 71: I don’t find the word approaches adequate in this context. I suggest to modify it in “mechanisms”**

**- Line 106-107: The new sentence “Nevertheless, this technology has not reached maturity hence unsatisfactory.” seems incomplete to me.**

**- I suggest to include some statistical results (at least p-values) in the abstract.**

Response: We modified all the minor concerns.
